# Single-cell RNA sequencing and AlphaFold 3 insights into cytokine signaling and its role in uveal melanoma

**DOI:** 10.3389/fimmu.2024.1458041

**Published:** 2025-01-23

**Authors:** Hongyan Sun, Cunzi Li, Zuhui Pu, Ying Lu, Zijing Wu, Lan Zhou, Hongzhan Lin, Yumo Wang, Tao Zi, Lisha Mou, Ming-ming Yang

**Affiliations:** ^1^ Department of Ophthalmology, Shenzhen People’s Hospital (The Second Clinical Medical College, Jinan University; The First Affiliated Hospital, Southern University of Science and Technology), Shenzhen, China; ^2^ Imaging Department, Shenzhen Institute of Translational Medicine, The First Affiliated Hospital of Shenzhen University, Shenzhen Second People’s Hospital, Shenzhen, China; ^3^ MetaLife Center, Shenzhen Institute of Translational Medicine, Shenzhen, Guangdong, China; ^4^ Post-doctoral Scientific Research Station of Basic Medicine, Jinan University, Guangzhou, China

**Keywords:** uveal melanoma, single-cell RNA sequencing, cytokine signaling, T cell, AlphaFold 3, Dictionary of Immune Responses to Cytokines

## Abstract

**Background:**

Uveal melanoma (UVM) is a form of eye cancer with a poor prognosis, particularly in metastatic patients. This study aimed to elucidate the cellular heterogeneity within UVM and identify prognostic biomarkers.

**Methods:**

We performed single-cell RNA sequencing (scRNA-seq) on primary and metastatic UVM samples. A UVM-specific gene signature was constructed using LASSO regression and validated via ROC curve analysis in the TCGA-UVM and GSE84976 cohorts. AlphaFold 3 was used to predict the 3D structures of key proteins. T-cell populations were analyzed using pseudotime trajectory mapping and interaction network visualization. CRISPR-Cas9 screening analysis was conducted to identify hub genes and cytokine pathways that may serve as therapeutic targets. Additionally, we constructed the Dictionary of Immune Responses to Cytokines at single-cell resolution to evaluate cytokine signatures.

**Results:**

ScRNA-seq revealed five major cell types within UVMs and subdivided them into seven distinct subtypes. Cytokine signaling analysis revealed differential expression of cytokine signaling in immune-related genes (CSIRGs) across these subtypes in primary and metastatic tumors. The UVM-specific gene signature demonstrated high predictive accuracy in ROC curve analysis and was associated with overall survival in Kaplan–Meier survival analyses. Additionally, AlphaFold 3 predicted the 3D structures of key proteins with high confidence. T-cell population analysis revealed complex developmental pathways and interaction networks in UVM. Myeloid-derived suppressor cells (MDSCs) were found to be increased in metastatic UVM, correlating with the enrichment of GM-CSF. CRISPR-Cas9 screening analysis identified hub genes and cytokine pathways with low gene effect scores across cell lines, indicating their potential importance in UVM.

**Conclusion:**

This study identified critical cellular subtypes and prognostic biomarkers in UVM, shedding light on targeted therapies. The insights into cytokine signaling and T-cell dynamics within the UVM microenvironment provide a foundation for developing personalized therapeutic strategies to improve patient outcomes.

## 1 Introduction

Uveal melanoma (UVM) has an incidence rate of approximately 5.1 per million in the US, with a mortality rate that remains high despite advances in treatment modalities (1). The primary treatment options for UVM include enucleation, radiotherapy, and laser therapy; however, these strategies often fail to prevent metastasis, which occurs in approximately 50% of patients (2). The aggressive nature and poor prognosis of metastatic UVM highlight the urgent need for novel diagnostic and therapeutic approaches (3).

Single-cell RNA sequencing (scRNA-seq) has revolutionized our understanding of the cellular heterogeneity within tumors, providing unprecedented insights into tumor microenvironments and their impact on disease progression (4, 5). ScRNA-seq has been particularly instrumental in identifying distinct cellular subpopulations and their functional states in various diseases, including diabetic retinopathy, melanoma, glioblastoma, and liver cancer (6–8). These studies have demonstrated that tumor heterogeneity significantly influences treatment response and patient outcomes, underscoring the importance of detailed cellular characterization in developing personalized therapeutic strategies (4, 9).

In UVM, the application of scRNA-seq reveal the complexity of the tumor microenvironment, shedding light on a variety of immune cell populations and their roles in tumor progression and immune evasion (10). For instance, recent studies have identified specific macrophage subsets with distinct functional properties that correlate with patient prognosis in patients with melanoma (11). Similarly, cytokine signaling pathways are important in shaping the tumor microenvironment and influencing tumor growth and metastasis (12, 13). These findings suggest that a comprehensive analysis of the cellular and molecular features of UVM could help therapeutic target identification.

We employed scRNA-seq to study the cellular composition and gene expression profiles of UVM samples. We identified major cell types and their subtypes. We also constructed the Dictionary of Immune Responses to Cytokines to evaluate cytokine signatures in immune cells within metastatic UVM compared to primary UVM. Moreover, we characterized cytokine signaling pathways, and constructed gene signatures based on cytokine signaling in immune-related genes (CSIRGs). We further validated these gene signatures in independent cohorts and performed survival analysis to assess their prognostic significance. Additionally, we utilized AlphaFold 3 to predict the three-dimensional structures of key proteins and conducted a detailed analysis of T-cell populations, including trajectory and interaction network analyses.

## 2 Methods

### 2.1 Single-cell RNA sequencing (scRNA-seq) analysis

To elucidate the cellular composition and functional characteristics of uveal melanoma (UVM), we analyzed scRNA-seq data on 8 primary and 3 liver metastatic UVM samples (GSE139829) (10). Quality control filters were applied to eliminate low-quality cells and potential doublets. Specifically, cells with high mitochondrial gene content (>10%) or an inappropriate gene count (fewer than 400 or more than 8000 genes) were excluded from further analysis. Analyses downstream were conducted using the Seurat (version 4.4.1) (14), including normalization, scaling, and dimensionality reduction. Data dimensionality was reduced using PCA. Batch effects were corrected using the ‘RunHarmony’ algorithm (15), and the t-SNE algorithm was employed to visualize the cellular distribution within each patient. Marker genes were used to identify major cell types in clusters (10). Further classification refined these major cell types into seven distinct subtypes by examining the expression profiles of specific markers. This detailed analysis provided a comprehensive understanding of the cellular heterogeneity within UVMs. Differentially expressed genes (DEGs) were identified by comparing primary UVM samples with metastatic samples using the Wilcoxon signed-rank test (adjusted P value <0.05).

### 2.2 Detailed ScRNA-seq analysis of T-cell populations in the UVM

#### 2.2.1 Cell type identification and marker analysis

T-cell subtypes were defined based on known marker genes, leading to the identification of eight main T-cell types.

#### 2.2.2 Pseudotime trajectory analysis

The Monocle package (version 2.32.0) was utilized to perform pseudotime trajectory analysis to explore the developmental hierarchy of T-cell subtypes.

#### 2.2.3 Interaction network mapping

Interaction networks among T-cell types were mapped to represent the number of ligand-receptor interactions by CellChat (version 1.5.0).

#### 2.2.4 Interaction strength analysis

The strength of interactions among various T-cell types was quantified, showing the weights or strengths of these interactions.

#### 2.2.5 Ligand–receptor interaction analysis

Detailed analysis of ligand-receptor interactions was performed to identify strong interactions between specific pairs of T-cell types.

### 2.3 Identification of myeloid-derived suppressor cells in scRNA-seq analysis

In our scRNA-seq analysis, we specifically included markers to identify MDSCs, which are known to play a significant role in tumor immune evasion and progression. We utilized *ITGAM (CD11b)*, *CD14*, and *CD33* as surface markers characteristic of MDSCs to distinguish this population from the scRNA-seq data. Additionally, we evaluated the expression levels of *PTGS2*, *S100A8*, *IL10*, *TGFB1*, and *VEGFA*, which are associated with the immunosuppressive function of MDSCs, to further understand their role within the tumor microenvironment. The identification and analysis of MDSCs were conducted using established bioinformatics pipelines, allowing us to accurately profile this cell population in both primary and metastatic UVM samples.

### 2.4 Cytokine signaling analysis

Immune-related gene set enrichment analysis with the irGSEA (version 3.3.2) package was performed to investigate the role of cytokine signaling in immune modulation. A density heatmap was generated to visualize the expression and distribution levels of cytokine signaling in immune-related genes (CSIRGs), accessed from the Reactome database (https://reactome.org), across different cell subtypes in primary and metastatic tumors. Higher enrichment scores are indicated by more intense red coloring.

### 2.5 Expression and distribution analysis

CSIRG module scores were calculated using AddModuleScore in Seurat (version 4.4.1) and generate a bubble plot displaying the expression and distribution levels of these genes across different cell subtypes within primary and metastatic tumors.

### 2.6 Construction of the dictionary of immune responses to cytokines for UVM

We utilized the Dictionary of Immune Responses to Cytokines at single-cell resolution to evaluate the cytokine signatures of various immune cells, specifically CD4+ T cells, CD8+ T cells, γδ T cells, Tregs, macrophages, and B cells, in metastatic UVM compared to primary UVM. This evaluation was based on the data collected by Cui et al., which measured transcriptional responses to individual cytokine stimulation. The cytokine signatures for each cell type in metastatic UVM were compared to those in primary UVM. Immune Response Enrichment Analysis (IREA) were performed (16). IREA Enrichment scores were calculated for each cytokine, identifying the top 10 cytokines with the strongest enrichment for each cell type.

### 2.7 Construction of gene signatures composed of CSIRGs for UVM

To construct robust gene signatures for UVM based on CSIRGs, we performed a comprehensive screening process across various cell types. Gene expression data from UVM samples were collected from TCGA-UVM (Training cohort, excluding 28 samples due to incomplete data. Used 52 complete samples for model) and GSE84976 (Testing cohort, including 28 samples) (17). The data preprocessing steps included normalization and log transformation to ensure comparability across samples. The intersection of CSIRGs and DEGs (identified by scRNA-seq analysis) was identified for each cell type to generate a refined list of candidate genes. Univariate Cox was performed to identify prognosis-related CSIRGs within each cell type. Genes significantly associated with patient prognosis (P < 0.05) were selected for further analysis. LASSO regression analysis was applied to further narrow the list of prognosis-related CSIRGs. LASSO regression imposes a penalty on the regression coefficients, effectively selecting the most relevant genes by decreasing less important genes to zero. Multivariate Cox was conducted on the genes selected from the LASSO analysis. This step ensured that the selected CSIRGs were independently associated with prognosis when considering other variables. The final gene signatures composed of CSIRGs were constructed for each cell type based on the results of the multivariate Cox.

### 2.8 Predictive accuracy and survival analysis of the CSIRG signature in UVM cohorts

To evaluate the predictive accuracy and prognostic significance of the CSIRG signature, we performed ROC curve analysis and Kaplan–Meier survival analysis in the TCGA-UVM and the GSE84976 cohorts. Using the CSIRG signature, risk scores were calculated for each sample in both cohorts. Risk scores were derived from a weighted sum of the expression levels of the CSIRG genes, where the weights corresponded to the regression coefficients from the multivariate Cox regression analysis. ROC was used to evaluate the predictive accuracy of the CSIRG signature in TCGA-UVM and GSE84976 cohorts. AUC was calculated to quantify the predictive performance of the CSIRG signature across different cell types.

Patients were stratified into CSIRG-high-risk and CSIRG-low-risk groups based on the median risk score. Kaplan–Meier survival curves were generated to compare overall survival between the CSIRG-high-risk and CSIRG-low-risk groups in both cohorts. Log-rank tests were used to determine the statistical significance of the differences in survival between the two groups.

The predictive power of the CSIRG signature was validated by comparing AUC values and survival outcomes in both the TCGA-UVM and GSE84976 cohorts. The consistency of the AUC values and survival trends between the cohorts was assessed to underscore the generalizability and robustness of the CSIRG signature.

#### 2.8.1 Survival status plot

The survival status of patients was plotted, with red dots representing deaths and green dots representing survival. This plot provides a visual representation of the mortality rate among CSIRG-high-risk and CSIRG-low-risk patients, highlighting the higher mortality rate in the CSIRG-high-risk group.

#### 2.8.2 Survival distribution plot

A survival distribution plot was generated to confirm the association between risk score and overall survival. The plot showed that higher risk scores were significantly associated with shorter overall survival. The above analyses were performed separately for T cells, B cells, fibroblasts, and tumor cells.

### 2.9 Protein structure prediction using AlphaFold 3

To gain insight into the structural characteristics of key proteins involved in UVM, we employed AlphaFold 3, a state-of-the-art protein structure prediction tool (18). The following steps outline the methodology used for predicting the three-dimensional structures of several critical proteins.

#### 2.9.1 Protein selection

We selected a set of proteins identified through LASSO-COX regression analysis, include MIF, PTGS2, ISG20, HMOX1, ABL2, LTBR, TNIP2, CD44, and FOXO3.

#### 2.9.2 AlphaFold 3 configuration

AlphaFold 3 was configured according to the standard protocol outlined in its user guide (18). The key parameters included the use of the latest model.

#### 2.9.3 Prediction procedure

Input Data Preparation: The primary amino acid sequences of the selected proteins were prepared and submitted to AlphaFold 3.

Prediction Execution: The model was run using default settings to predict the structures. Each protein prediction was performed multiple times to ensure accuracy and reliability.

Confidence Metrics: Confidence scores, including pLDDT (per-residue confidence) and pTM (predicted template modeling) scores, were calculated to evaluate the reliability of the predicted structures. A pTM score above 0.5 indicates a fold change similar to that of the true structure, while scores above 0.8 indicate high-quality predictions.

#### 2.9.4 Protein structure validation using experimental data

We have undertaken the validation of AlphaFold 3 predicted protein structures by cross-referencing with experimentally determined structures available in the Protein Data Bank (PDB). Specifically, for the protein ISG20, we accessed the PDB entry 1WLJ, which provides the crystal structure of human ISG20 complexed with two Mn2+ ions and uridine 5’-monophosphate (UMP) at a resolution of 1.9 Å. The PDB entry, deposited by Horio, T., offers a comprehensive structural framework for our validation process (PDB DOI: https://doi.org/10.2210/pdb1WLJ/pdb) (19).

The structure of ISG20, classified as a HYDROLASE, was determined using X-ray crystallography and is derived from the organism Homo sapiens, with the protein expressed in Escherichia coli. There are no noted mutations in the deposited structure. We compared the specific structural features, including alpha-helices, beta-strands, and other secondary structures predicted by AlphaFold 3, with the features observed in the PDB structure 1WLJ. This comparison allowed us to assess the accuracy of the predicted secondary and tertiary structures and provided a solid foundation for validating the reliability of our in silico predictions. Using the experimental data from PDB entry 1WLJ, we were able to confirm the structural accuracy of our AlphaFold 3 predictions for ISG20, thereby strengthening the validity of our findings and their relevance to the study of UVM.

### 2.10 Analysis of gene effect scores

We analyzed gene effect scores derived from CRISPR-Cas9 screening data for eight uveal melanoma cell lines—MEL202, MEL270, MEL285, MEL290, OMM25, UPMD1, UPMM3 and WM3772F—using the DepMap R package (version 3.20) (20). Our investigation focused on a total of 9 hub genes (*MIF*, *PTGS2*, *ISG20*, *HMOX1*, *ABL2*, *LTBR*, *TNIP2*, *CD44*, and *FOXO3*), complemented by 7 additional genes known to be involved in cytokine pathways (*CD40*, *CD40LG*, *CSF2*, *IL12A*, *IL12B*, *IL12RB1*, and *IL12RB2*). This comprehensive analysis aims to identify potential therapeutic targets within the context of UVM.

### 2.11 Statistical analysis

All analyses were conducted using R (version 4.3.1). A P value of less than 0.05 was considered to indicate statistical significance unless otherwise specified.

## 3 Results

### 3.1 Single-cell analysis of uveal melanoma (UVM)

**Figure 1:**
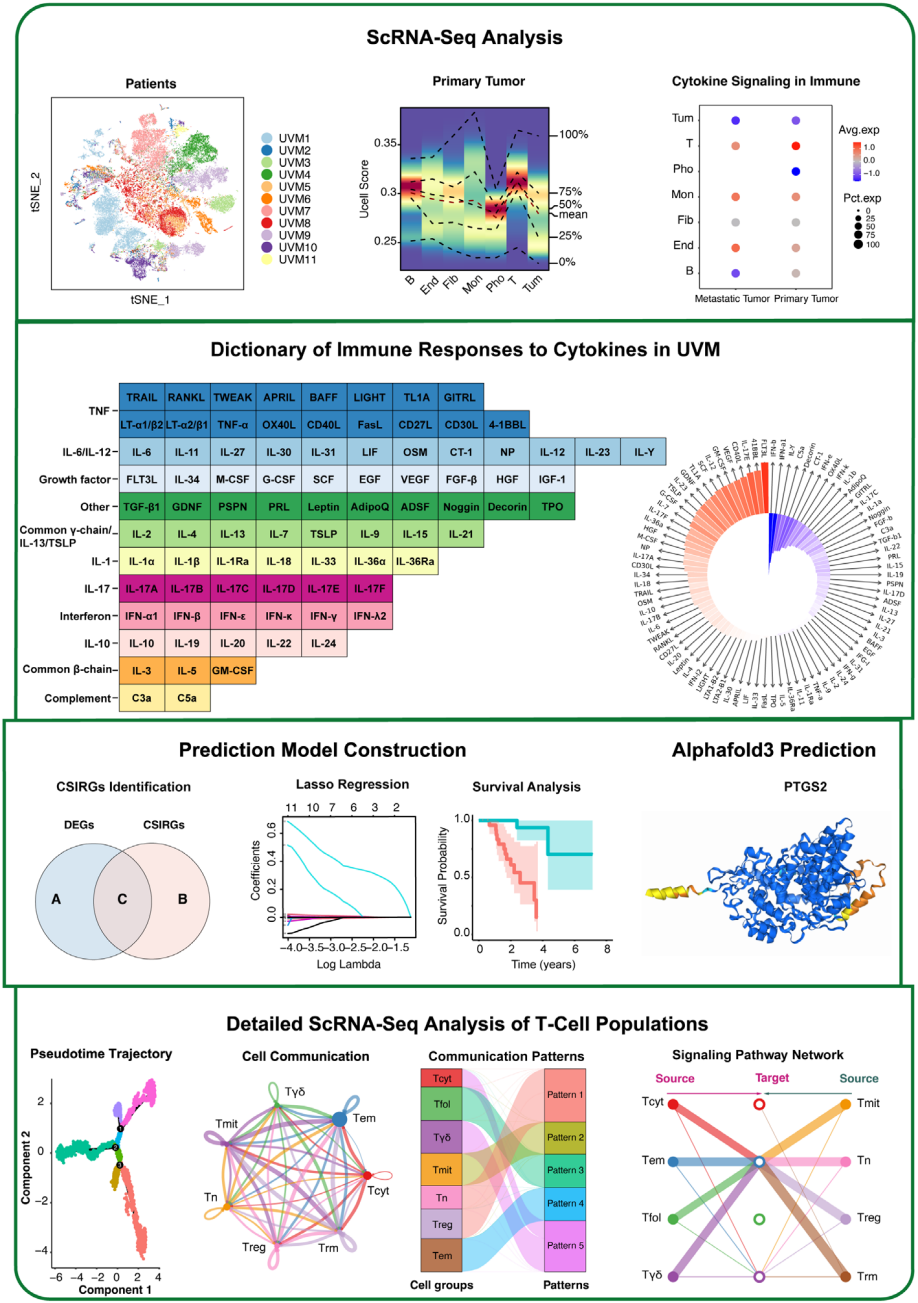
Workflow of this study.

The workflow is shown in **Figure 1**. To elucidate the cellular composition and functional characteristics of UVM, we performed single-cell RNA sequencing (scRNA-seq) analysis of primary and metastatic UVM samples. We utilized t-SNE plots to visualize the cellular distribution and identify five major cell types, further refining these into seven distinct subtypes. Marker expression profiles were examined to characterize each subtype. To investigate cytokine signaling, irGSEA and Seurat’s AddModuleScore were used, revealing differential expression patterns of cytokine signaling genes. We then constructed robust gene signatures based on cytokine signaling in immune-related genes (CSIRGs) across various cell types by intersecting CSIRGs with DEGs, followed by univariate Cox, LASSO, and multivariate Cox regression analyses. The predictive accuracy and survival relevance of these gene signatures were validated using ROC curve analysis and Kaplan–Meier survival curves in two independent cohorts (the TCGA-UVM and GSE84976 cohorts), confirming the robustness of the signatures. Additionally, AlphaFold 3 was used to predict the three-dimensional structures of key proteins involved in UVM. Finally, detailed scRNA-seq analysis of T-cell populations, including pseudotime trajectory analysis and interaction network mapping, was performed to understand T-cell dynamics and interactions within the UVM microenvironment.

**Figure 2:**
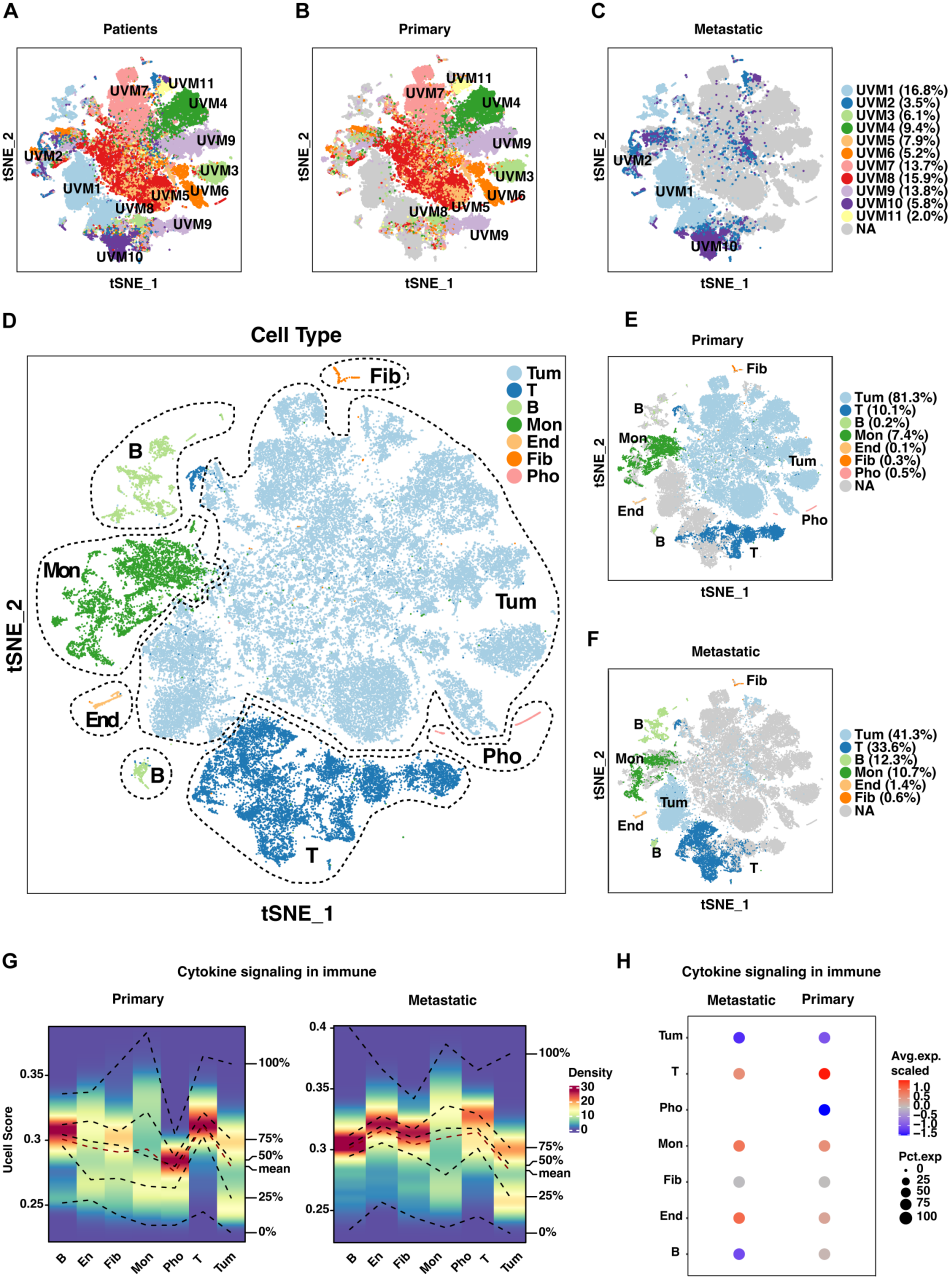
Single-cell analysis of uveal melanoma (UVM). **(A)** T-SNE plots depict the distribution of single cells from primary and metastatic UVM samples across patients. Separate T-SNE plots for **(B)** 8 primary and **(C)** 3 metastatic UVM patients highlight cellular heterogeneity. **(D)** Cell types are further classified into seven distinct subtypes based on unique gene expression profiles. Distribution of these subtypes is compared between **(E)** primary and **(F)** metastatic UVM patients, revealing subtype-specific differences. **(G)** A density heatmap illustrates cytokine signaling in immune-related gene (CSIRG) expression and distribution, with higher enrichment scores indicated by intensified red in both primary and metastatic tumors. **(H)** A bubble plot, generated with AddModuleScore in Seurat, visualizes the expression and distribution of CSIRGs across cell subtypes, providing insights into immune cell dynamics within tumors. Tum, Tumor; End, Endothelial; Fib, Fibroblast; Mon, monocyte and macrophage; Pho, Photoreceptor.

**Figure 3:**
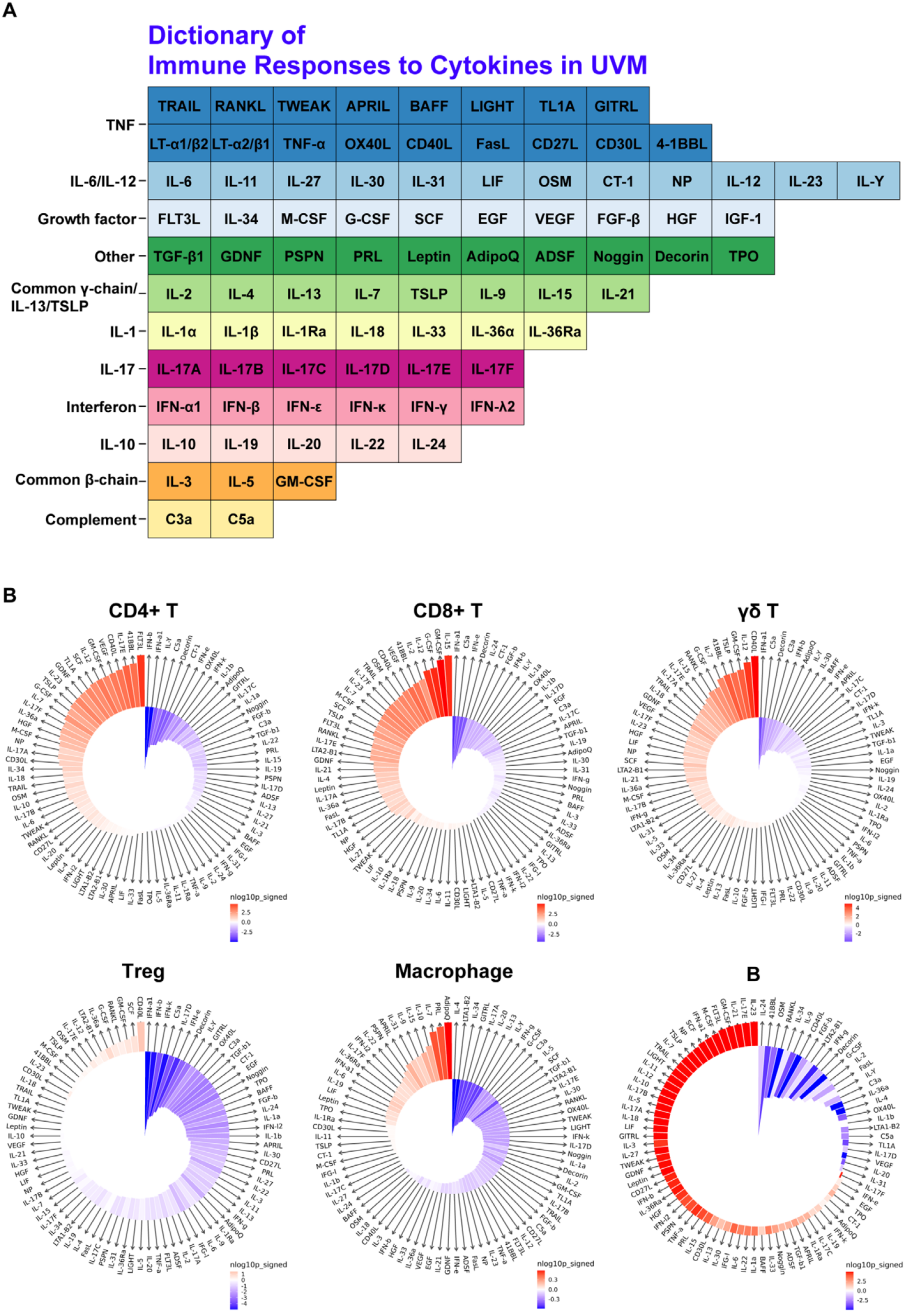
Cytokine signature enrichment in immune cells. **(A)** Dictionary of Immune Responses to Cytokines in UVM. A total of 86 cytokines were analyzed to compare their responses in metastatic UVM versus primary UVM. **(B)** IREA Cytokine Enrichment Plot. This plot shows the enrichment score (ES) for each of the 86 cytokine responses in CD4+ T cells, CD8+ T cells, γδ T cells, Tregs, macrophages, and B cells in metastatic UVM compared to primary UVM. The bar length represents the ES, and the shading indicates the FDR-adjusted P value (two-sided Wilcoxon rank-sum test), with darker colors representing more significant enrichment (red indicates enrichment in metastatic UVM, blue indicates enrichment in primary UVM).

To elucidate the cellular composition and functional characteristics of UVM, we performed scRNA-seq analysis on primary and metastatic UVM samples. The t-SNE plot (**Figure 2A**) illustrates the distribution of single cells from primary and metastatic UVM samples within each patient. Our dataset includes samples from 11 patients, comprising 8 with primary UVM (**Figure 2B**) and 3 with metastatic UVM (**Figure 2C**). Metastatic UVM patients (UVM1, UVM2, UVM10) have cell counts ranging from 2085 to 9949, with proportions varying from 3.5% to 16.8% (**Table 1**). Primary UVM patients (UVM3 to UVM9) have cell counts ranging from 1171 to 9438, with proportions varying from 2.0% to 15.9% (**Table 1**).

**Table 1:** The cell counts and proportion of each patient.

Patient	Category	Cell Count	Proportion
UVM1	Metastatic	9949	16.8%
UVM2	Metastatic	2085	3.5%
UVM3	primary	3597	6.1%
UVM4	primary	5581	9.4%
UVM5	primary	4673	7.9%
UVM6	primary	3072	5.2%
UVM7	primary	8131	13.7%
UVM8	primary	9438	15.9%
UVM9	primary	8193	13.8%
UVM10	Metastatic	3415	5.8%
UVM11	primary	1171	2.0%

We identified five major cell types through marker analysis, with tumor cells and leukocytes being the most prevalent (**Supplementary Figures S1A-C**). In metastatic UVM, leukocytes have the highest proportion (14.8%), while photoreceptor cells have a proportion of 0. In primary UVM, tumor cells have the highest proportion (60.2%), followed by leukocytes (13.1%).

Further classification refined these major cell types into seven distinct subtypes, providing a more granular understanding of the cellular heterogeneity in UVM (**Figures 2D–F**). In metastatic UVM, T cells have the highest proportion among leukocytes (8.8%), followed by B cells (3.2%). In primary UVM, T cells have the highest proportion among leukocytes (7.5%), followed by monocytes and Macrophages (5.9%).

We examined the marker expression profiles for each of the seven cell subtypes, which revealed distinct expression patterns that characterized each subtype (**Supplementary Figure S1D**). These profiles are essential for identifying specific cellular functions and interactions within the tumor microenvironment.

To explore the impact of cytokine signaling on immune modulation in UVM, we conducted irGSEA analysis. A density heatmap depicts the expression and distribution of CSIRGs across various cell subtypes in primary and metastatic tumors (**Figure 2G**). Notably, the heatmap reveals higher enrichment scores in subtypes with active cytokine signaling, visualized by intensified red shading. A bubble plot further elucidates the differential expression patterns of CSIRGs, underscoring the distinct cytokine signaling profiles between primary and metastatic tumor environments (**Figure 2H**). Collectively, our single-cell analysis delineates the intricate cellular landscape and dynamic cytokine signaling in UVM, identifying potential therapeutic targets and enhancing our comprehension of the tumor microenvironment.

### 3.2 Construction of the dictionary of immune responses to cytokines for UVM

In constructing the Dictionary of Immune Responses (16) to Cytokines for UVM, we analyzed single-cell data to compare the cytokine signatures of various immune cell populations in metastatic versus primary UVM (**Figures 3A, B**). Utilizing data from Cui et al., we identified the top 10 enriched cytokines for each cell type in metastatic UVM. Notably, GM-CSF was dominantly enriched across all T cell types and B cells in metastatic UVM. Similarly, CD40L and IL12 were consistently enriched in metastatic UVM for all T cell subtypes. These findings underscore the significant alterations in cytokine signaling associated with metastatic progression (**Figure 3B**).

### 3.3 Construction of gene signatures composed of CSIRGs for UVM

**Figure 4:**
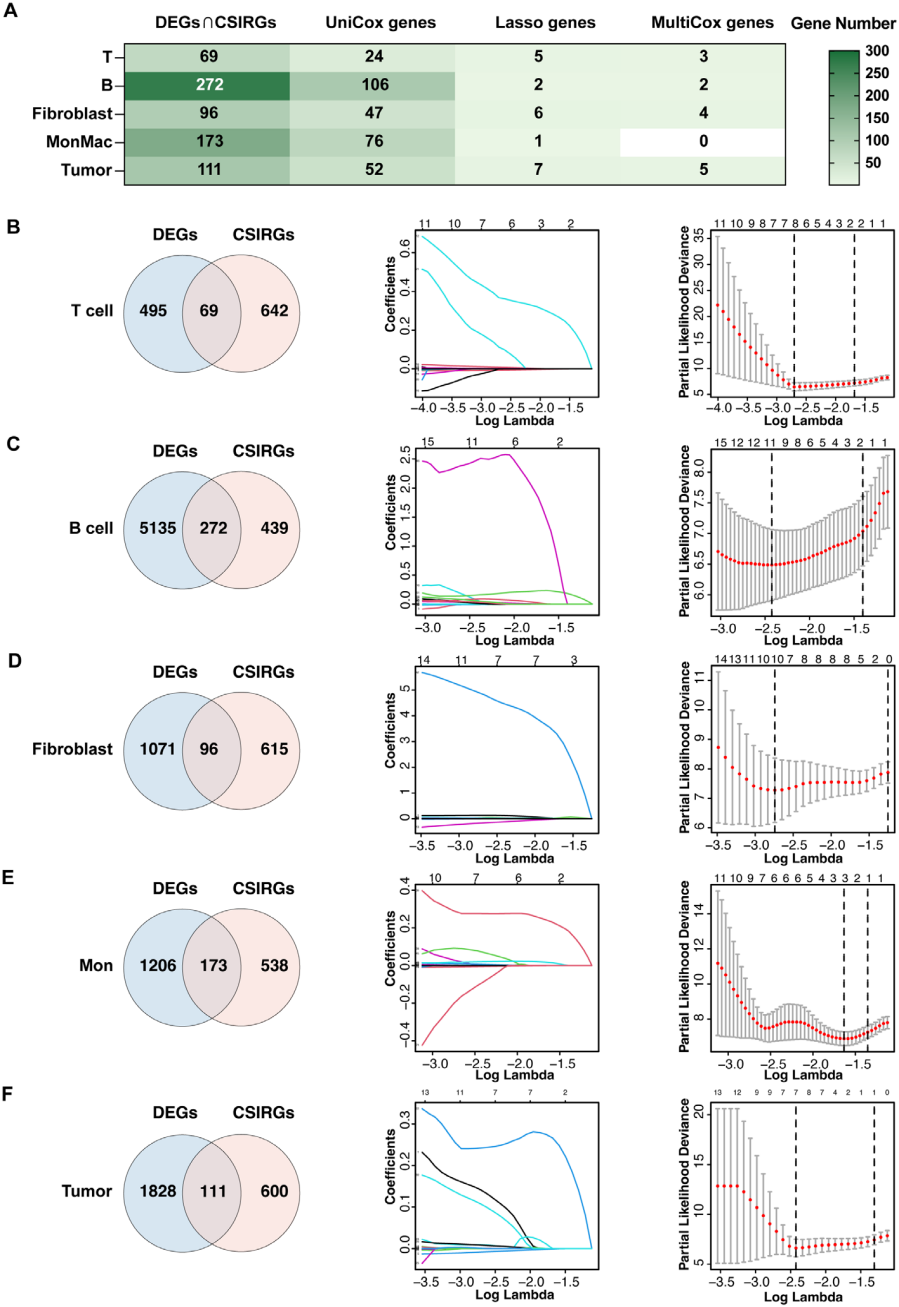
Construction of gene signatures composed of CSIRGs for UVM. **(A)** The screening process of CSIRGs in different cell types. The detailed steps included **(B)** screening of T cells, **(C)** screening of B cells, **(D)** screening of fibroblasts, **(E)** screening of monocyte and macrophages, and **(F)** screening of tumor cells. The process involved the following steps: first, identifying the intersection of CSIRGs and differentially expressed genes (DEGs) between primary and metastatic UVM tumors; second, performing univariate Cox regression analysis to identify prognosis-related CSIRGs; third, applying LASSO regression analysis to further narrow the prognosis-related CSIRGs; and fourth, conducting multivariate Cox regression analysis to finalize the gene signatures. Mon, monocyte and macrophage.

To construct robust gene signatures for UVM based on CSIRGs, we performed a comprehensive screening process across major cell types, including T cells, B cells, fibroblasts, monocytes and macrophages, and tumor cells (**Figure 4A**). By intersecting CSIRGs with differentially expressed genes (DEGs), we identified a refined list of candidate genes for each cell type (**Figures 4B–F**). Following this, we performed univariate Cox regression analyses to determine the prognostic significance of these genes. Using LASSO regression analysis to further refine the prognosis-related CSIRGs and reduce the risk of overfitting. Finally, we performed multivariate Cox regression analysis to construct the prognostic model. The final gene signatures, confirmed through rigorous screening, provide valuable prognostic biomarkers and potential therapeutic targets for UVM. Specifically, we identified the following CSIRGs: for T cells, *CD44*, *ISG20*, and *MIF*; for B cells, *ISG20* and *PTGS2*; for fibroblasts, *PTGS2*, *HMOX1*, *ABL2*, and *FOXO3*; and for tumor cells, *CD44*, *ISG20*, *ABL2*, *MIF*, and *TNIP2*. These hub genes, including *MIF*, *PTGS2*, *ISG20*, *HMOX1*, *ABL2*, *LTBR*, *TNIP2*, *CD44*, and *FOXO3*, will be the focus of our subsequent analyses.

### 3.4 Predictive accuracy and survival analysis of the CSIRG signature in UVM cohorts

**Figure 5:**
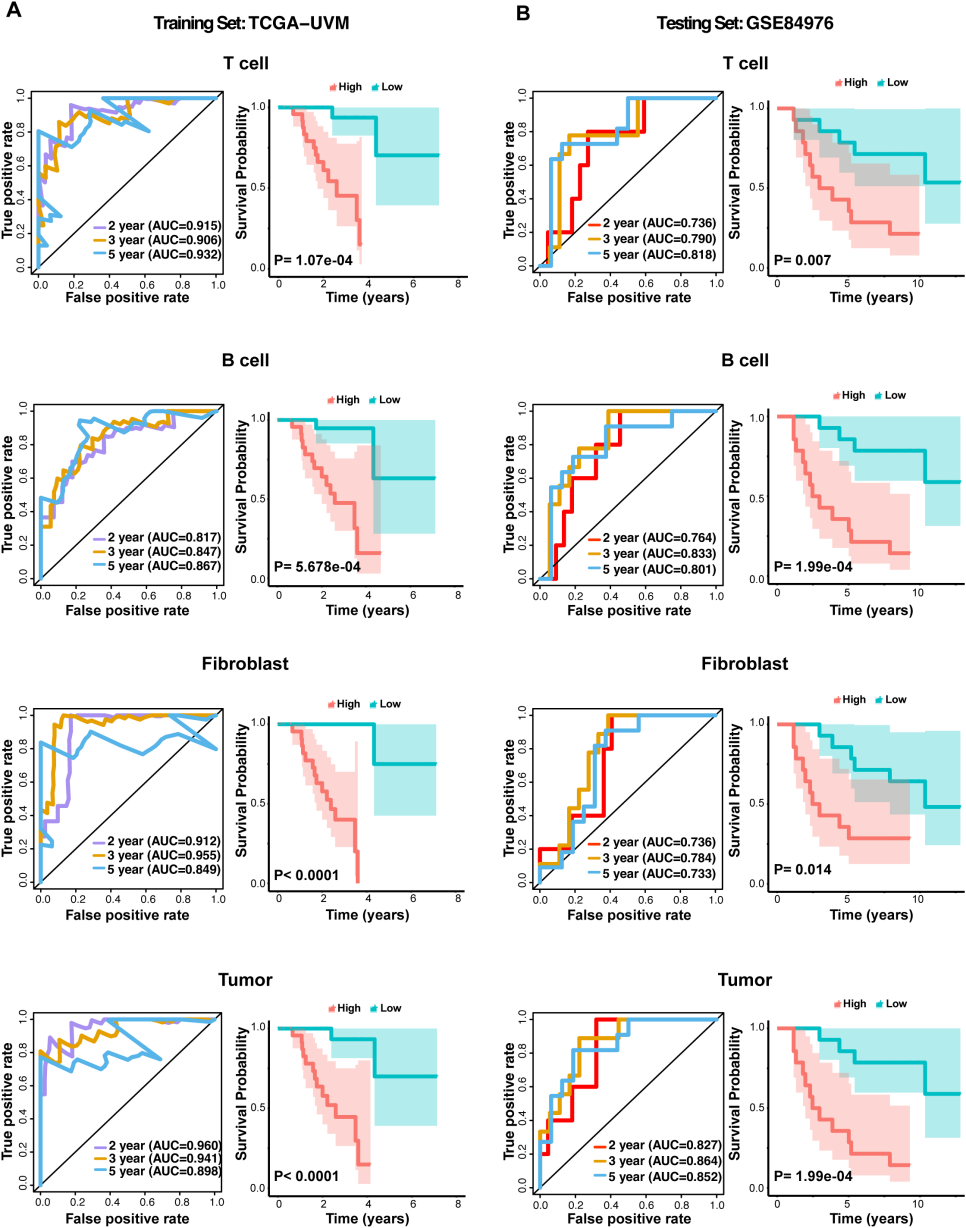
ROC curves and Kaplan–Meier survival analysis for the CSIRG signature in the UVM cohort. **(A)** ROC curves evaluating the predictive accuracy of the CSIRG signature in the TCGA-UVM cohort, including T cells, B cells, fibroblasts, and tumor cells. **(B)** ROC curves evaluating the predictive accuracy of the CSIRG signature for T cells, B cells, fibroblasts, and tumor cells in the GSE84976 cohort. Kaplan–Meier survival curves comparing overall survival between the CSIRG-high-risk and CSIRG-low-risk groups in both cohorts highlighted significant differences in survival outcomes.

We evaluated the predictive accuracy of the CSIRG signature in UVM using ROC curve analysis across two independent cohorts: TCGA-UVM and GSE84976. The TCGA-UVM cohort’s ROC curves validated the signature’s predictive accuracy for various cell types, with AUC values attesting to its robustness in predicting overall survival (**Figure 5A**). The GSE84976 cohort corroborated these findings, showing similar AUC values and reinforcing the signature’s broad applicability (**Figure 5B**). Kaplan-Meier survival analysis revealed that, in both cohorts, the CSIRG-high-risk group had significantly lower overall survival compared to the CSIRG-low-risk group, highlighting the signature’s prognostic value (**Figures 5A, B**).

### 3.5 Survival analysis for evaluating the accuracy of CSIRG signature in UVM

To validate the prognostic accuracy of the CSIRG signature, we performed survival analysis across different cell types in UVM samples. Risk score distribution revealed clear distinctions between low- and high-CSIRG-risk groups, with higher risk scores associated with greater mortality and shorter overall survival. This pattern was consistent across T cells, B cells, fibroblasts, and tumor cells (**Supplementary Figures S2A–D**). These results validate the robust prognostic utility of the CSIRG signature for predicting overall survival in UVM patients and highlight its potential for clinical risk stratification and management.

### 3.6 Protein structure prediction using AlphaFold 3

**Figure 6:**
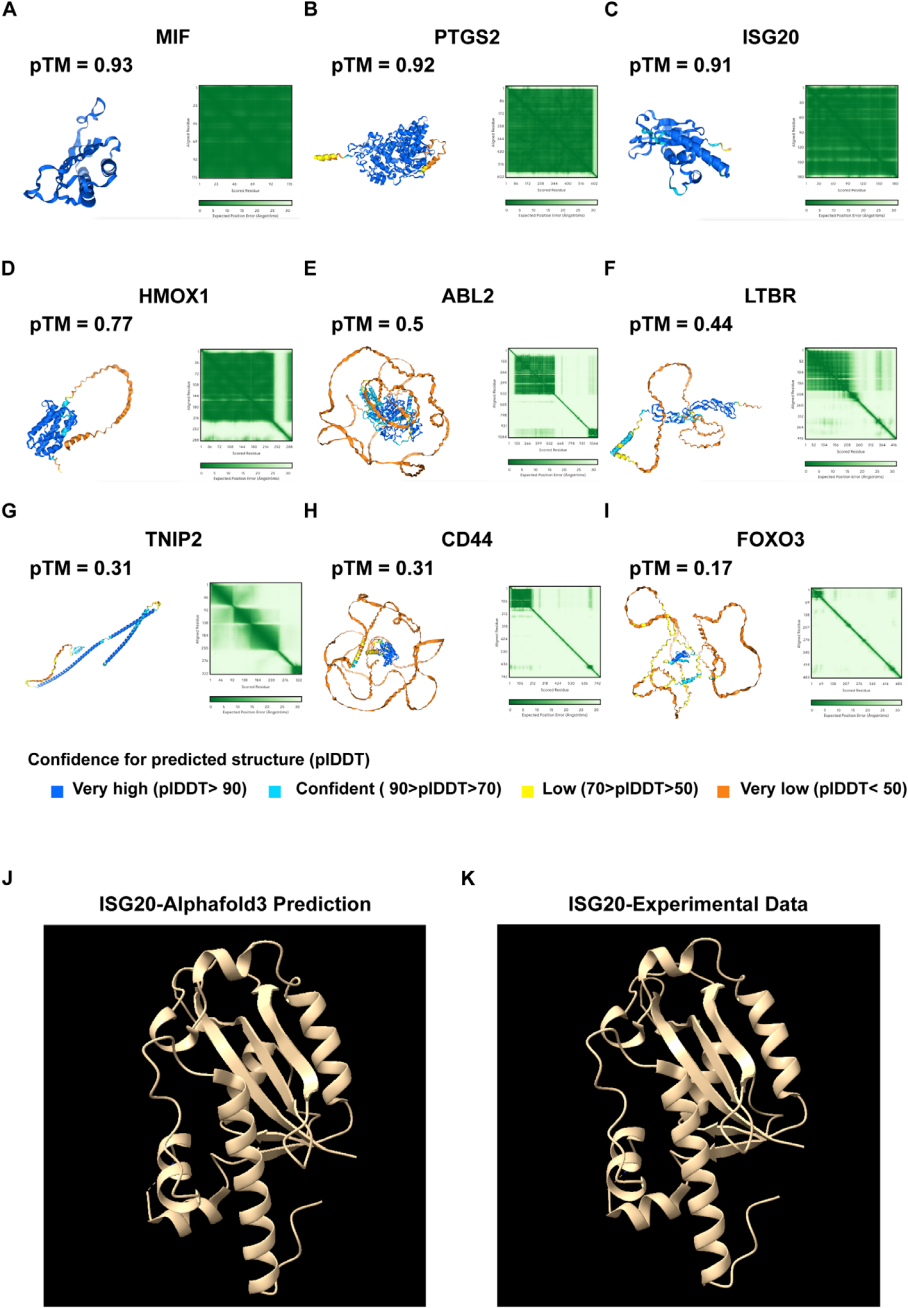
Protein structure prediction using AlphaFold 3. **(A)** Predicted protein structure of MIF, with a pTM score of 0.93. **(B)** Predicted protein structure of PTGS2, with a pTM score of 0.92. **(C)** Predicted protein structure of ISG20, with a pTM of 0.91. **(D)** Predicted protein structure of HMOX1, with a pTM of 0.77. **(E)** Predicted protein structure of ABL2, with a pTM score of 0.5. **(F)** Predicted protein structure of the LTBR, with a pTM score of 0.44. **(G)** Predicted protein structure of TNIP2, with a pTM score of 0.31. **(H)** Predicted protein structure of CD44, with a pTM score of 0.31. **(I)** Predicted protein structure of FOXO3, with a pTM score of 0.17. pTM score: The predicted template modeling (pTM) score is derived from the template modeling (TM) score, which measures the accuracy of the entire structure. A pTM score above 0.5 indicates that the overall predicted fold for the complex might be similar to the true structure. Comparative analysis of **(J)** AlphaFold 3-predicted ISG20 structure with **(K)** experimental data.

To elucidate the structural characteristics of key proteins involved in UVM, we used AlphaFold 3 to predict the three-dimensional structures of several important proteins. These proteins, identified through LASSO-COX regression analysis, include MIF, PTGS2, ISG20, HMOX1, ABL2, LTBR, TNIP2, CD44, and FOXO3 (**Figures 6A–I**). By predicting their structures, we aim to gain insights into their functional roles within the disease context and explore their potential as therapeutic targets. The pTM scores indicate model accuracy. MIF (pTM 0.93), PTGS2 (pTM 0.92), and ISG20 (pTM 0.91) exhibit distinct folds and active sites. HMOX1 (pTM 0.77), ABL2 (pTM 0.5), LTBR (pTM 0.44), TNIP2 (pTM 0.31), CD44 (pTM 0.31), and FOXO3 (pTM 0.17) highlight domains crucial for their functions.

We have undertaken the validation of AlphaFold 3 predictions by cross-referencing with existing structural data in the Protein Data Bank (PDB). Focusing on ISG20 as a case study, we meticulously examined the predicted structural features, including alpha-helices, beta-strands, and other secondary structures, and compared them with the corresponding features observed in PDB structures. This comparative analysis was instrumental in validating the accuracy of the predicted secondary and tertiary structures. Our findings revealed a high degree of accuracy in the AlphaFold 3 prediction, as evidenced by the alignment with experimental data (**Figures 6J, K**). These predictions enhance our understanding of their roles in UVM and can guide targeted therapy development.

### 3.7 T cell phenotype and interactions in primary and metastatic lesions

**Figure 7:**
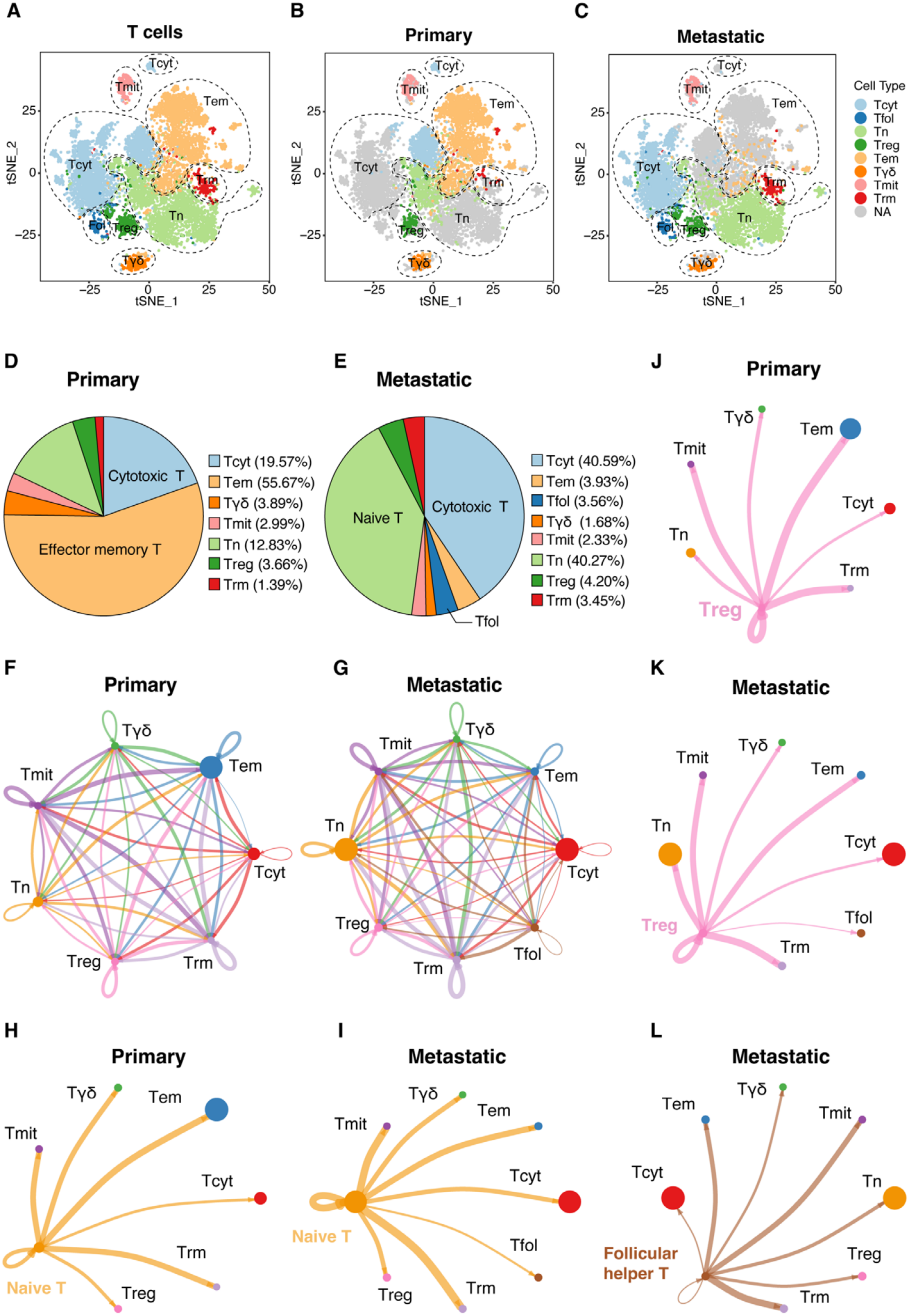
Detailed ScRNA-seq analysis of T-cell populations. **(A)** Cellular clusters of T cells were identified in primary and metastatic UVMs, showing the eight main cell types within the tumor samples. Separate t-SNE plots for **(B)** primary and **(C)** metastatic UVM patients highlight cellular heterogeneity. The cell proportion of eight cell types for **(D)** primary and **(E)** metastatic UVM. Interaction network among T cells for **(F)** primary and **(G)** metastatic UVM, representing the number of ligand–receptor interactions among various T-cell types, highlighting network complexity. Interactions between naive T cells and other subtypes for **(H)** primary and **(I)** metastatic UVM. Interactions between Treg cells and other subtypes for **(J)** primary and **(K)** metastatic UVM. Interactions between Follicular helper T cells and other subtypes for **(L)** metastatic UVM. Tcyt, Cytotoxic T; Tfol, Follicular helper T; Tn, Naive T; Treg, Regulatory T; Tem, Effector memory T; Tγδ, γδ T; Tmit, Mitotic T; Trm, Resident memory T.

Our detailed scRNA-seq analysis provided comprehensive insights into T-cell populations within primary and metastatic UVM. We identified eight main T-cell types, distinguished by unique gene expression profiles, and characterized their functional roles within the tumor microenvironment (**Figures 7A–C**). Notably, metastatic UVM showed a higher proportion of cytotoxic and naive T cells compared to primary UVM, which was predominantly characterized by effector memory T cells. Follicular helper T cells, crucial for B cell activation, were exclusively identified in metastatic UVM (**Figures 7D, E**). Pseudotime trajectory analysis revealed a developmental pathway from naive T cells to effector memory and cytotoxic T cells, highlighting dynamic differentiation processes in UVM (**Supplementary Figures S3A, B**).

Mapping the interaction network among T-cell types underscored the complexity of communication pathways regulating T-cell function within the tumor microenvironment (**Figures 7F, G**). Analysis of the T cell interaction network disclosed substantial changes in cellular communication between primary and metastatic tumors. Notably, there was an increase in interactions between naive T cells and other subtypes (**Figures 7H, I**), along with augmented interactions between regulatory T cells (Tregs) and other subtypes within metastatic lesions (**Figures 7J, K**). Exclusively in metastatic UVM, interactions between follicular helper T cells and other subtypes were identified (**Figure 7L**), suggesting a role in immune evasion mechanisms.

### 3.8 Cell communication patterns and signaling pathways

Our scRNA-seq and computational analyses uncovered distinct T-cell communication patterns and signaling pathways in the UVM microenvironment (**Supplementary Figures S4A–H**). Naive and Tregs mainly use pattern 1 (ICAM, IL16), while mitotic and follicular helper T cells use patterns 2 (CD70, BAG, PECAM1) and 3 (CXCL, BTLA), respectively. Resident memory T cells and γδ T cells communicate via patterns 4 and an unidentified pattern 5.

Effector memory T cells, mitotic T cells, and resident memory T cells primarily receive ligand stimulation through pattern 1 (MHC-I, LCK, VCAM, CXCL, CD70, CD137, PECAM1). γδ T cells and Treg cells receive stimuli through patterns 2 and 3 (CLEC, BAG, MHC-II, SELPLG, IL16). Naive T cells mainly receive stimuli through pattern 4 (ITGB2, ADGRE5).

The autocrine and paracrine signaling of key pathways, including MHC-I, VCAM, CXCL, ADGRE5, CLEC, and LCK, impact various T-cell subtypes and modulate signaling within mitotic T, naive T, Treg, and resident memory T cells. The visualizations in **Supplementary Figures S4C–H** depict the complexity of UVM’s communication networks, suggesting potential therapeutic targets to improve patient outcomes.

### 3.9 Distribution and frequency of Myeloid-derived Suppressor Cells in UVM

**Figure 8:**
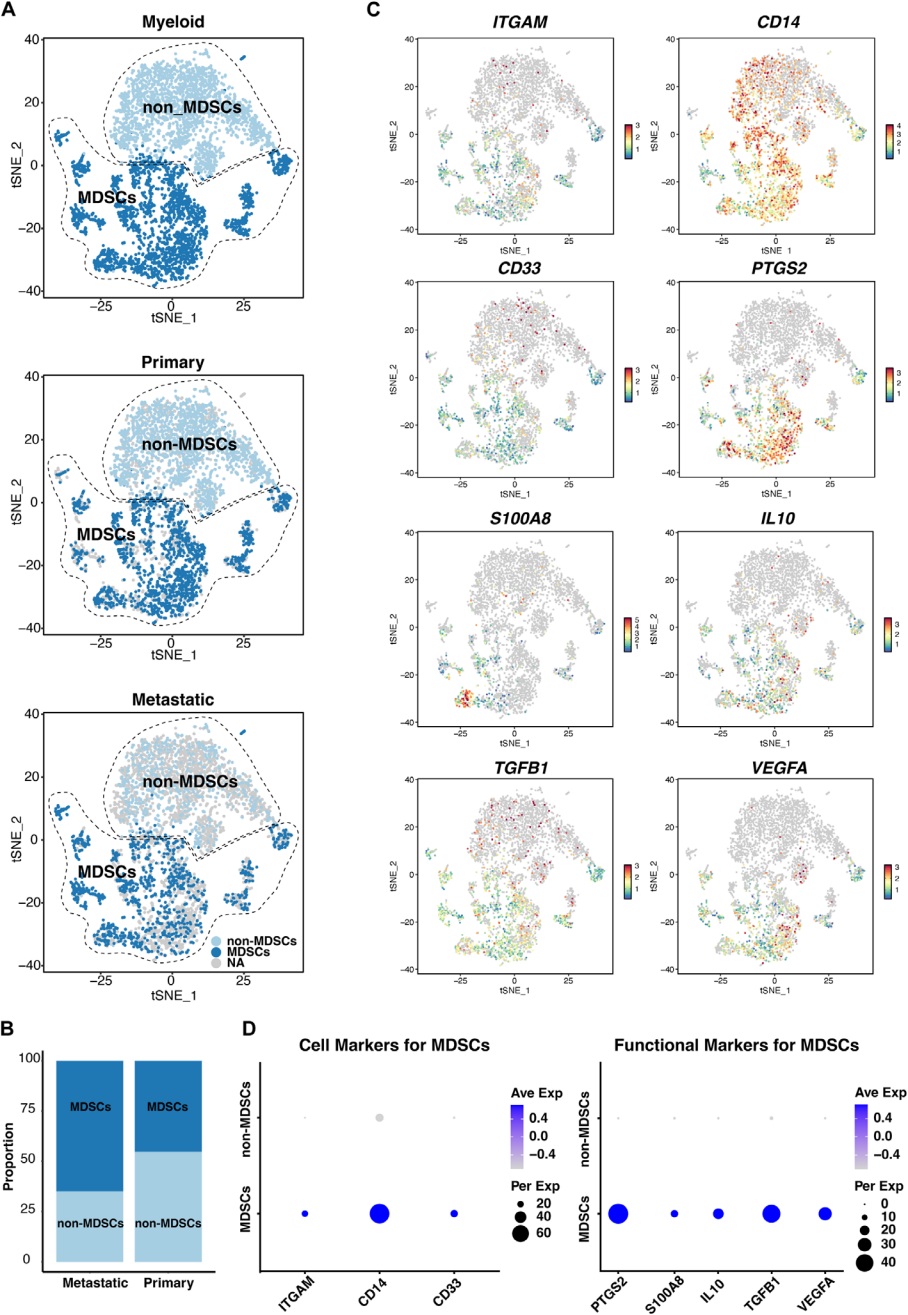
ScRNA-seq analysis of myeloid populations in UVM. **(A)** Identification of myeloid-derived suppressor cells (MDSCs), and non-MDSCs across primary and metastatic UVM samples, revealing distinct myeloid subpopulations. **(B)** Comparative distribution of MDSCs and non-MDSCs in primary versus metastatic UVM, showcasing a shift in myeloid composition associated with metastasis. **(C)** T-SNE plots and **(D)** dot plots display the expression levels of MDSC markers *ITGAM* (*CD11b*), *CD14*, and *CD33*, along with functional markers *S100A8*, *IL10*, *TGFB1*, *VEGFA*, and *PTGS2*, providing insights into the immunomodulatory capacity of MDSCs in UVM.

Our analysis revealed a notable increase in the frequency of MDSCs (**Figures 8A, B**), as identified by *ITGAM* (*CD11b*), *CD14*, and *CD33* (**Figures 8C, D**), in metastatic UVM samples compared to primary tumors. This increase in MDSCs correlates with the observed dominant enrichment of GM-CSF in metastatic tumors (**Figure 3B**), suggesting a potential link between MDSC accumulation and the pro-tumorigenic cytokine environment. Furthermore, the expression analysis of *PTGS2*, *S100A8*, *IL10*, *TGFB1* and *VEGFA* indicated a functional shift in the immune landscape (**Figures 8C, D**), possibly contributing to the immunosuppressive phenotype observed in metastatic lesions.

### 3.10 CRISPR-Cas9 screening reveals hub genes and cytokine pathways in UVM: implications for therapeutic targeting

**Figure 9:**
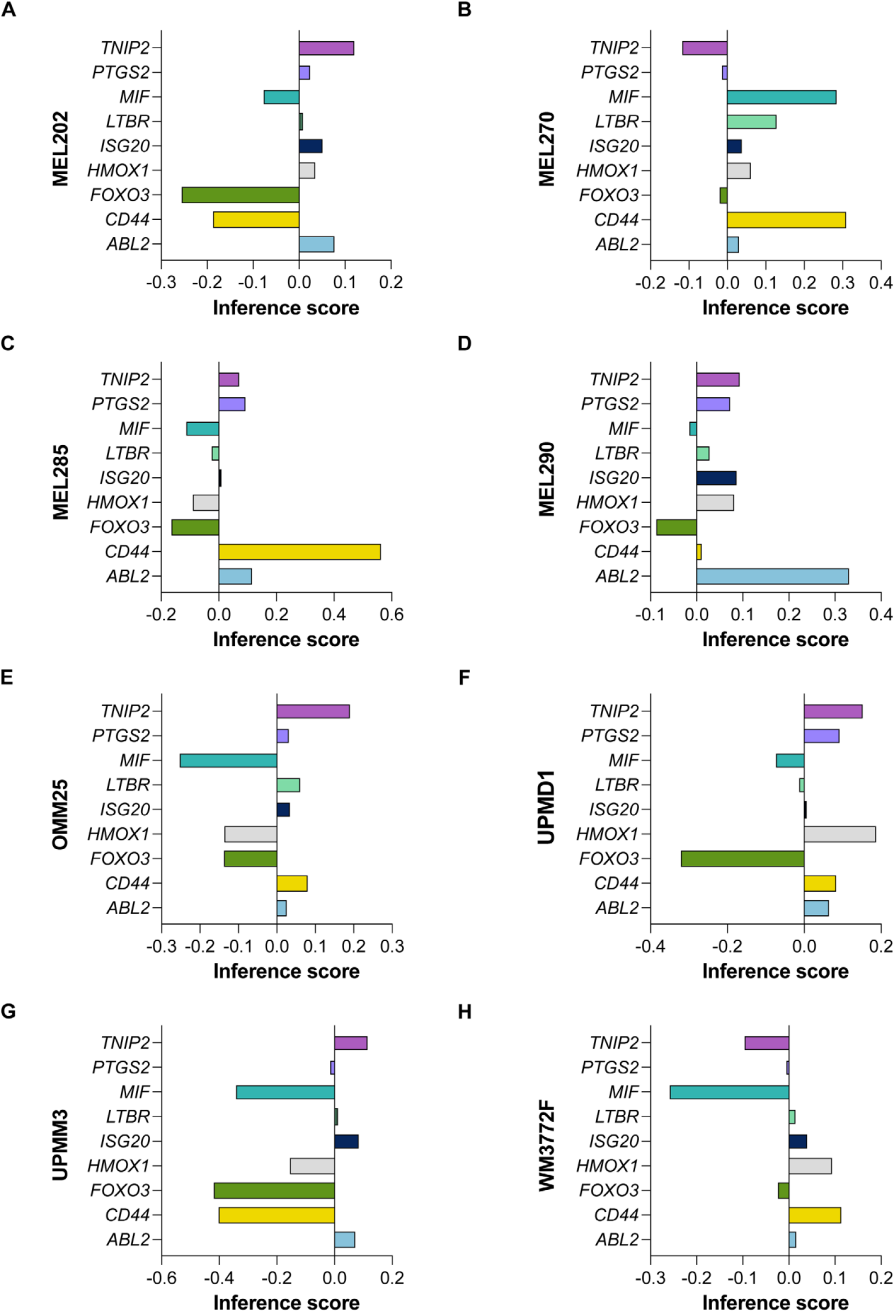
CRISPR-Cas9 Screening Identifies Hub Genes in UVM. The gene effect scores of nine hub genes (*MIF*, *PTGS2*, *ISG20*, *HMOX1*, *ABL2*, *LTBR*, *TNIP2*, *CD44*, and *FOXO3*) derived from CRISPR-Cas9 knockout screening in eight UVM cell lines. **(A)** MEL202, **(B)** MEL270, **(C)** MEL285, **(D)** MEL290, **(E)** OMM25, **(F)** UPMD1, **(G)** UPMM3 and **(H)** WM3772F.

**Figure 10:**
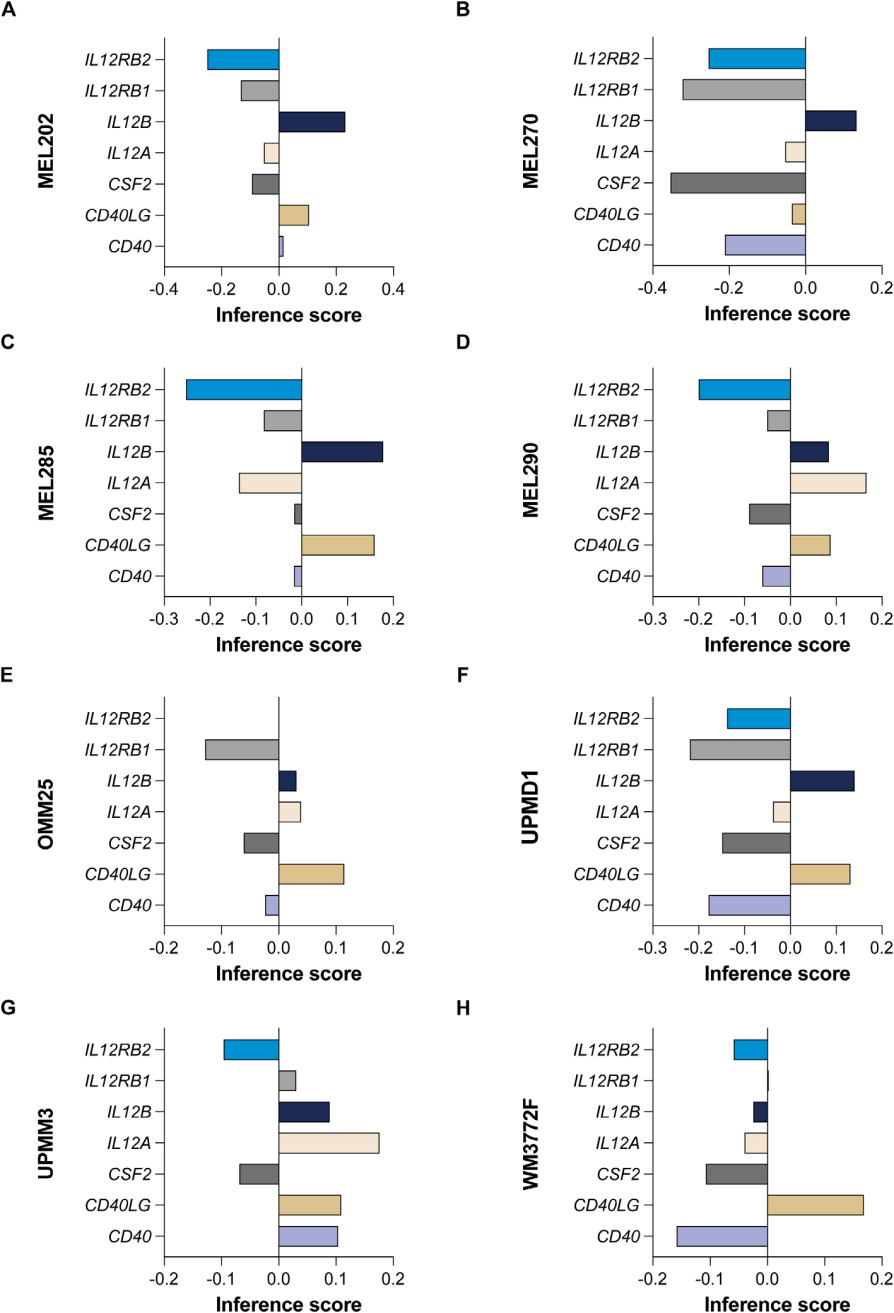
CRISPR-Cas9 screening reveals cytokine pathway genes in UVM. The gene effect scores for seven additional genes implicated in cytokine pathways (*CD40*, *CD40LG*, *CSF2*, *IL12A*, *IL12B*, *IL12RB1*, and *IL12RB2*) in eight UVM cell lines. **(A)** MEL202, **(B)** MEL270, **(C)** MEL285, **(D)** MEL290, **(E)** OMM25, **(F)** UPMD1, **(G)** UPMM3 and **(H)** WM3772F.

Utilizing gene effect scores derived from CRISPR-Cas9 knockout screening, we conducted an analysis of hub genes and cytokine pathways that may significantly contribute to UVM and potentially serve as therapeutic targets. A total of 9 hub genes (*MIF*, *PTGS2*, *ISG20*, *HMOX1*, *ABL2*, *LTBR*, *TNIP2*, *CD44*, and *FOXO3*) (**Figures 9A–H**), along with 7 additional genes implicated in cytokine pathways (*CD40*, *CD40LG*, *CSF2*, *IL12A*, *IL12B*, *IL12RB1*, and *IL12RB2*) (**Figures 10A–H**).

Our analysis revealed that the gene effect scores for *FOXO3*, *CSF2*, and *IL12RB2* were consistently low across all eight cell lines, suggesting a potential dependency on these genes for cellular viability. Similarly, the gene effect score for *MIF* was low in the majority of the cell lines (seven out of eight), indicating its potential importance in UVM. Furthermore, *IL12RB1* and *CD40* exhibited low scores in nearly all cell lines (six out of eight), highlighting their possible roles in the disease process. These findings underscore the potential of these genes as therapeutic targets in UVM and warrant further investigation.

## 4 Discussion

Uveal melanoma (UVM) with a high risk of metastasis, particularly to the liver, significantly impacts patient survival and quality of life (21, 22). Despite advances in local tumor control, the prognosis for patients with metastatic UVM remains poor, with limited effective systemic therapies available (2). Therefore, there is an urgent need to understand the molecular and cellular mechanisms underlying UVM to develop more effective diagnostic and therapeutic strategies.

To determine UVM tumor cellular heterogeneity, we used single-cell RNA sequencing (scRNA-seq), focusing on cytokine signaling, gene expression, and distribution. Our analysis identified major cell types and their subtypes, providing a comprehensive overview of the cellular landscape in primary and metastatic UVM. The significant cytokine enrichment observed in various immune cell types underscores the complexity of the tumor microenvironment and highlights potential therapeutic targets.

Our findings indicate that certain cytokines, such as CD40L, IL12, and GM-CSF, are strongly enriched in metastatic UVM compared to primary UVM across multiple immune cell types. This suggests a prominent role for these cytokines in promoting tumor progression and metastasis. Specifically, CD40L and IL12 were consistently enriched in CD4+ T cells, CD8+ T cells, γδ T cells, and Treg cells in metastatic UVM, pointing to their importance in modulating immune responses within the tumor microenvironment. In Treg cells, cytokines such as SCF, GM-CSF, RANKL, G-CSF, IL36a, LTA2-B1, IL12, IL17E, and OSM were also found to be strongly enriched in metastatic UVM. This enrichment indicates a potential shift in the regulatory landscape that may facilitate tumor immune evasion and persistence. Similarly, macrophages in metastatic UVM showed strong enrichment for cytokines like Adiponectin, Prolactin, IL7, IL10, IL15, IL9, IL31, APRIL, Persephin, and IL22, highlighting their role in creating a pro-tumorigenic environment through immune modulation and support of tumor growth. B cells in metastatic UVM were enriched for cytokines such as IL23, IL17E, IL21, GM-CSF, Flt3L, M-CSF, IFNa1, SCF, Neuropoietin, and TSLP. This suggests an active involvement of B cells in the metastatic process, potentially through cytokine-mediated interactions that enhance tumor cell survival and dissemination.

The consistent enrichment of GM-CSF across all T cell types and B cells in metastatic UVM further emphasizes its pivotal role in the tumor microenvironment. GM-CSF is known to modulate the functions of various immune cells, promoting inflammation and potentially facilitating tumor progression. Its dominant presence in metastatic UVM underscores its potential as a therapeutic target. These results highlight the critical role of cytokine signaling in shaping the immune landscape of metastatic UVM. The identification of key cytokines enriched in metastatic UVM provides valuable insights into the mechanisms driving tumor progression and immune modulation. These findings shed light on the development of targeted therapies aimed at disrupting these cytokine-mediated interactions, thereby improving patient outcomes in UVM. Future studies should focus on further elucidating the functional roles of these cytokines and their potential as therapeutic targets in UVM.

By constructing gene signatures based on cytokine signaling in immune-related genes (CSIRGs) and validating their prognostic significance, we aimed to identify potential biomarkers and therapeutic targets. Our comprehensive analysis, including protein structure prediction and detailed T-cell population analysis, provides novel insights into the tumor microenvironment and immune landscape of UVM. These findings hold promise for improving the diagnosis, prognosis, and treatment of UVM, ultimately enhancing patient outcomes.

Our findings revealed distinct expression patterns of CSIRGs in primary versus metastatic UVM samples, suggesting that these pathways may contribute to tumor evolution and metastatic potential. The differential expression of CSIRGs, such as macrophage migration inhibitory factor (*MIF*) and prostaglandin-endoperoxide synthase 2 (*PTGS2*), which were further validated through AlphaFold 3 structural predictions, underscores their potential as therapeutic targets.

*MIF* is a proinflammatory cytokine (23), that has been implicated in various malignancies, including melanoma, where it contributes to tumor progression and metastasis through its effects on cell proliferation, angiogenesis, and immune evasion (24–26). In our study, we utilized AlphaFold 3 to predict the three-dimensional structure of *MIF*, achieving a high pTM score of 0.93, indicating that this was a reliable structural model. The differential expression of *MIF* in various cell subtypes within UVM, as revealed by our scRNA-seq analysis, underscores its significance in the tumor microenvironment. Targeting *MIF* could modulate the immune landscape of UVM, suggesting a novel approach for therapeutic intervention.

*PTGS2*, also known as COX-2, which are key mediators of inflammation and pain (27, 28). *PTGS2* is frequently overexpressed in various cancers, including melanoma, and is associated with poor prognosis due to its role in promoting tumor growth, angiogenesis, and immune suppression (29, 30). Our study utilized AlphaFold 3 (18) to predict the structure of PTGS2, yielding a high pTM score of 0.92, which provides a reliable model for further functional and therapeutic studies. The elevated expression of *PTGS2* in specific UVM cell subtypes, as identified through our scRNA-seq analysis, highlights its potential as a biomarker and therapeutic target.

Our study revealed significant differential expression of CSIRGs across various cell subtypes in UVM through irGSEA and Seurat’s AddModuleScore function. Cytokine signaling pathways play crucial roles in modulating immune responses, inflammation, and cell proliferation and are pivotal in cancer progression and metastasis. For instance, the interleukin-6 (IL-6) signaling pathway has been implicated in promoting tumor growth and immune evasion in various cancers (31–34). Similarly, the interferon-gamma (IFN-γ) pathway is known for its role in enhancing antitumor immunity and has been associated with a better prognosis in melanoma patients (35–37).

The construction of a CSIRG-based gene signature and its validation through ROC curve analysis and Kaplan–Meier survival curves further highlight the prognostic value of these pathways. The high AUC values in both the TCGA-UVM and GSE84976 cohorts indicate that our gene signature is a robust predictor of overall survival in UVM patients. This suggests that targeting CSIRGs could improve therapeutic outcomes and provide a basis for personalized treatment strategies.

The validation of AlphaFold 3 predictions against existing PDB data serves a dual purpose: it not only substantiates the precision of our predicted structures but also enhances our comprehension of the molecular mechanisms at play in UVM. By aligning our predicted structures with PDB data, we are poised to refine our predictive methodologies, thereby augmenting the precision of future structural biology investigations in this domain. Looking ahead, future studies that incorporate experimental validation techniques such as cryo-electron microscopy (cryo-EM) and X-ray crystallography will be pivotal in further corroborating the accuracy of AlphaFold 3 predictions.

The structural predictions obtained through AlphaFold 3 offer several advantages for understanding the roles of these proteins in UVM. Firstly, they allow for the identification of functional sites that are essential for protein activity. Secondly, they facilitate the mapping of interaction networks and pathways implicated in UVM. Thirdly, these structures are invaluable for the rational design of therapeutic agents targeting UVM-associated proteins. Lastly, they provide mechanistic insights into protein function, which is crucial for developing targeted therapies.

The distinct T cell phenotypes and interaction patterns observed between primary and metastatic UVM lesions provide valuable insights into the tumor’s immunological heterogeneity. The higher proportion of cytotoxic T cells in metastatic lesions may suggest an initial immune response against the tumor, which is subsequently countered by the tumor’s immune evasion mechanisms, as evidenced by the increased interactions involving Tregs and the absence of follicular helper T cells in primary tumors.

These differences in T cell interactions and phenotypes between primary and metastatic UVM lesions underscore the dynamic and complex nature of the immunological response in the tumor microenvironment. The increased expression of exhaustion markers in metastatic lesions may reflect a tumor-mediated mechanism to dampen effective anti-tumor immunity, facilitating metastatic spread. The altered interaction networks and differential expression of cytokine receptors on T cells between primary and metastatic tumors indicate a shift in T cell responsiveness to cytokine signaling, which may be crucial for tumor progression.

These findings emphasize the necessity for targeted immunotherapies that can address the specific immunological challenges presented by metastatic UVM. Understanding the phenotypic and interaction changes in T cells between primary and metastatic lesions may lead to the development of more effective therapeutic strategies, such as enhancing the anti-tumor immune response or disrupting the immunosuppressive network in metastatic tumors.

The increased presence of myeloid-derived suppressor cells (MDSCs), in metastatic UVM, as identified by CD11b, CD14, and CD33, underscores their potential role in facilitating tumor progression and immune evasion. The correlation between MDSC frequency and GM-CSF enrichment suggests a complex interplay between these cells and the cytokine milieu, which may be pivotal in the metastatic cascade. Our findings are consistent with the liver being the most common metastatic site for UVM, as confirmed by the metastatic samples in our study. The liver’s immunosuppressive environment may provide a fertile ground for MDSCs to exert their suppressive functions, thereby promoting tumor metastasis and survival.

The functional assessment of MDSCs through the analysis of *PTGS2, S100A8*, *IL10*, *TGFB1*, and *VEGFA* expression provides a comprehensive view of their immunomodulatory activities within the tumor microenvironment. These insights highlight the potential of targeting MDSCs or their associated cytokines as a therapeutic strategy to combat UVM metastasis. Future studies will focus on elucidating the functional roles of these MDSC-associated genes and their potential as therapeutic targets in UVM.

Our analysis revealed a significant increase in CD40L expression in metastatic UVM compared to primary tumors. This elevated expression of CD40L, typically found on activated T cells and crucial for T cell activation, suggests a complex role in the immune modulation and evasion strategies employed by metastatic tumors. The higher levels of CD40L may indicate an immunosuppressive environment that is detrimental to effective anti-tumor immunity, thereby facilitating metastatic spread. This is in line with previous studies that highlight the dual role of CD40/CD40L in tumor biology, where membrane-bound CD40L can lead to tumor clearance, while soluble CD40L (sCD40L) promotes tumor survival by hindering apoptosis, suppressing the immune system, and promoting tumor angiogenesis (38).

The increased CD40L expression in metastatic lesions may also reflect altered T cell functions within the metastatic microenvironment, with potential implications for tumor progression and immune cell interactions. This could imply that T cells in metastatic lesions are more activated or have a different activation profile compared to those in primary tumors, potentially due to differences in the local cytokine milieu or other microenvironmental factors. Furthermore, sCD40L inhibits the immune system through various mechanisms, including the induction of MDSCs and regulatory T cells, while also increasing the expression of interleukin-10 (38). These effects suppress antigen presentation, cytokine production, macrophage activation, and antigen-specific T cell proliferation (38). Additionally, sCD40L inhibits the production of IL-12 by activated monocytes and upregulates the expression of programmed cell death-1 (PD-1), a key regulator of T cell exhaustion (38).

In the tumor microenvironment, sCD40L, especially when present at high levels, has a stronger immunosuppressive effect. This suggests that the elevated CD40L expression in metastatic UVM might contribute to the enhancement of tumor cell survival, proliferation, and invasion, possibly through interactions with other cells in the tumor microenvironment. Further studies are warranted to explore the functional consequences of CD40L upregulation in metastatic UVM and its potential as a therapeutic target. Understanding these mechanisms could provide valuable insights into the development of targeted therapies aimed at disrupting these cytokine-mediated interactions, thereby improving patient outcomes in UVM.

Our study provides significant clinical implications for the management of UVM by identifying novel biomarkers and therapeutic targets. The identification of key cytokines such as CD40L, IL12, and GM-CSF, which are strongly enriched in metastatic UVM, offers potential targets for immunotherapeutic intervention. For instance, the dominant enrichment of GM-CSF in metastatic tumors suggests that it could be a promising target for therapeutic antibodies or small molecule inhibitors, potentially improving patient outcomes by disrupting the immunosuppressive tumor microenvironment. This approach differs from existing methods and could offer more precise targeting of UVM cells, as suggested by our comprehensive analysis of cytokine signaling in UVM.

Compared to the study by Durante et al. (10), which focused on the novel subclonal genomic complexity and transcriptional states of tumor cells in UVM, our study provides a comprehensive analysis of the immune landscape and cytokine signaling in UVM. We have identified key cytokines enriched in metastatic UVM, which were not reported in their study. This adds a new dimension to our understanding of UVM pathogenesis and suggests that immune modulation could be a viable therapeutic strategy, complementing the genetic insights provided by Durante et al.

In contrast to Li et al. (11), who investigated the role of macrophage subsets in UVM, our study expands on this by examining the broader immune context, including T cells, and potential for combination therapies. Our findings of distinct cytokine signatures and their enrichment in metastatic tumors present a novel mechanism that could be targeted, which was not explored in their research. By identifying cytokines such as CD40L and IL12, which are enriched in various T cell subtypes in metastatic UVM, we provide a basis for developing targeted immunotherapies that could disrupt these cytokine-mediated interactions and improve patient outcomes in UVM.

Finally, our study evaluates UVM cytokine signaling pathways comprehensively, revealing their critical roles in tumor progression and patient prognosis. This research identified major cell types and subtypes within UVM, highlighted the differential expression and distribution of immune-related genes across various cell subtypes, and constructed prognostic gene signatures based on CSIRGs, which were validated for their predictive accuracy in multiple cohorts. Additionally, this study provided detailed insights into T-cell populations and predicted the 3D structures of key proteins using AlphaFold 3. In addition to offering insights into the molecular landscape of UVM and presenting biomarkers for prognosis and therapeutic targets. Future research should focus on validating these findings through wet laboratory experiments and clinical trials to enhance their applicability in clinical practice.

## Data Availability

The original contributions presented in the study are included in the article/**Supplementary Material**. Further inquiries can be directed to the corresponding authors.

## Supplementary material

Supplementary Figure 1
**(A)** T-SNE plot showing five major cell types identified five predominant cell types in uveal melanoma (UVM) through marker analysis, with tumor cells and leukocytes being the most abundant. Separate T-SNE plots for **(B)** primary and **(C)** metastatic UVM patients highlight cellular heterogeneity. **(D)** Expression profiles of the markers for each of the seven major cell types. Tum, Tumor; Leu, Leukocytes; End, Endothelial; Fib, Fibroblast; Pho, Photoreceptor ; Mon, monocyte and macrophage.

Supplementary Figure 2 Survival Analysis for Evaluating the Accuracy of the Gene Signature. **(A)** T cells: Distribution of risk scores between the CSIRG-high-risk and CSIRG-low-risk groups (left) and survival status of UVM patients in the CSIRG-high-risk and CSIRG-low-risk groups (right). Red dots represent CSIRG-high-risk patients who died, whereas green dots represent CSIRG-low-risk patients who survived. The survival distribution plot indicates that higher risk scores are associated with shorter overall survival. **(B)** B cells: Distribution of risk scores between the CSIRG-high-risk and CSIRG-low-risk groups (left) and survival status of UVM patients in the CSIRG-high-risk and CSIRG-low-risk groups (right). **(C)** Fibroblasts: Distribution of risk scores between the CSIRG-high-risk and CSIRG-low-risk groups (left) and survival status of UVM patients in the CSIRG-high-risk and CSIRG-low-risk groups (right). **(D)** Tumor cells: Distribution of risk scores between the CSIRG-high-risk and CSIRG-low-risk groups (left) and survival status of UVM patients in the CSIRG-high-risk and CSIRG-low-risk groups (right). The survival distribution plot indicates that higher risk scores are associated with shorter overall survival.

Supplementary Figure 3
**(A)** Pseudotime trajectory analysis of T cell subtypes utilizing the Monocle package, illustrating the ordering of T cells along a primary developmental trajectory with three distinct bifurcations. **(B)** The analysis revealed that the developmental hierarchy of T cells, commencing with naive T cells and advancing toward effector memory T cells and cytotoxic T cells, as determined by the pseudotime trajectory analysis.

Supplementary Figure 4 Cell Communication Patterns and Signaling Pathways. **(A)** The communication patterns of outgoing cells in UVM, with flow widths indicating the contribution of each element to the pattern. Patterns 1-5 are represented, with specific pathways associated with naive T, regulatory T, mitotic T, follicular helper T, resident memory T, and γδ T cells. **(B)** The communication patterns of incoming cells in UVM, highlighting the pathways through which effector memory T, mitotic T, and resident memory T cells receive ligand stimulation, as well as γδ T and Treg cells. **(C-H)** the autocrine and paracrine signaling of key pathways (MHC-I, VCAM, CXCL, ADGRE5, CLEC, LCK) in T cells, with left panels showing the impact on various T-cell subtypes and right panels depicting signaling modulation among mitotic T, naive T, Treg, and resident memory T cells. Circle sizes and line widths correspond to cell numbers and communication strength, respectively. Abbreviations: Tcyt, Cytotoxic T; Tfol, Follicular helper T; Tn, Naive T; Treg, Regulatory T; Tem, Effector memory T; γδ T, Tγδ; Tmit, Mitotic T; Trm, Resident memory T.
