# Supplementary figures and images for: Single-cell RNA sequencing and AlphaFold 3 insights into cytokine signaling and its role in uveal melanoma
Source: Front Immunol. 2025 Jan 23;15:1458041. doi: 10.3389/fimmu.2024.1458041 (PMC11798937; doi:10.3389/fimmu.2024.1458041)

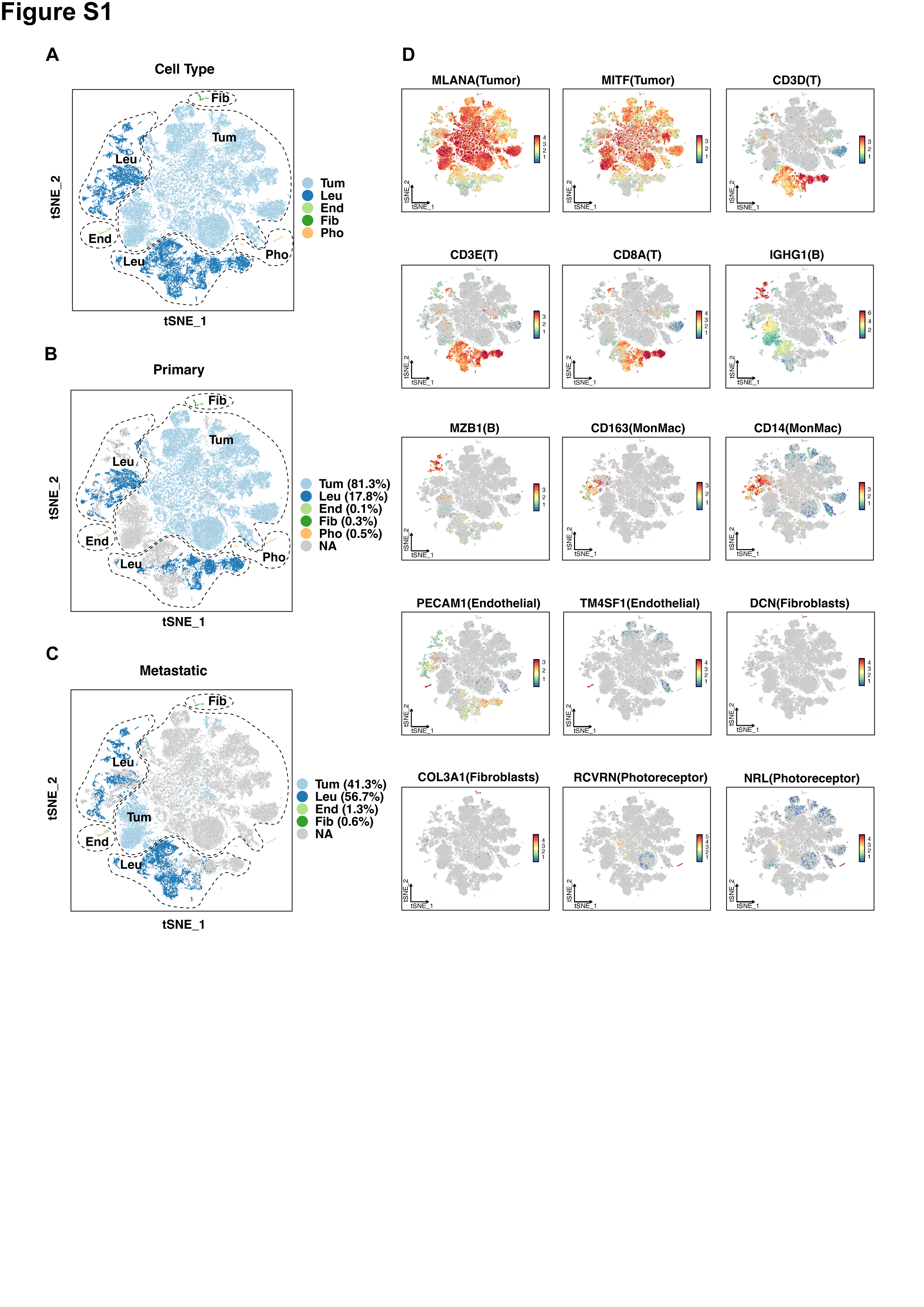

Supplement: Supplementary Figure 1 — (A) T-SNE plot showing five major cell types identified five predominant cell types in uveal melanoma (UVM) through marker analysis, with tumor cells and leukocytes being the most abundant. Separate T-SNE plots for (B) primary and (C) metastatic UVM patients highlight cellular heterogeneity. (D) Expression profiles of the markers for each of the seven major cell types. Tum, Tumor; Leu, Leukocytes; End, Endothelial; Fib, Fibroblast; Pho, Photoreceptor ; Mon, monocyte and macrophage. [file Image1.tif]

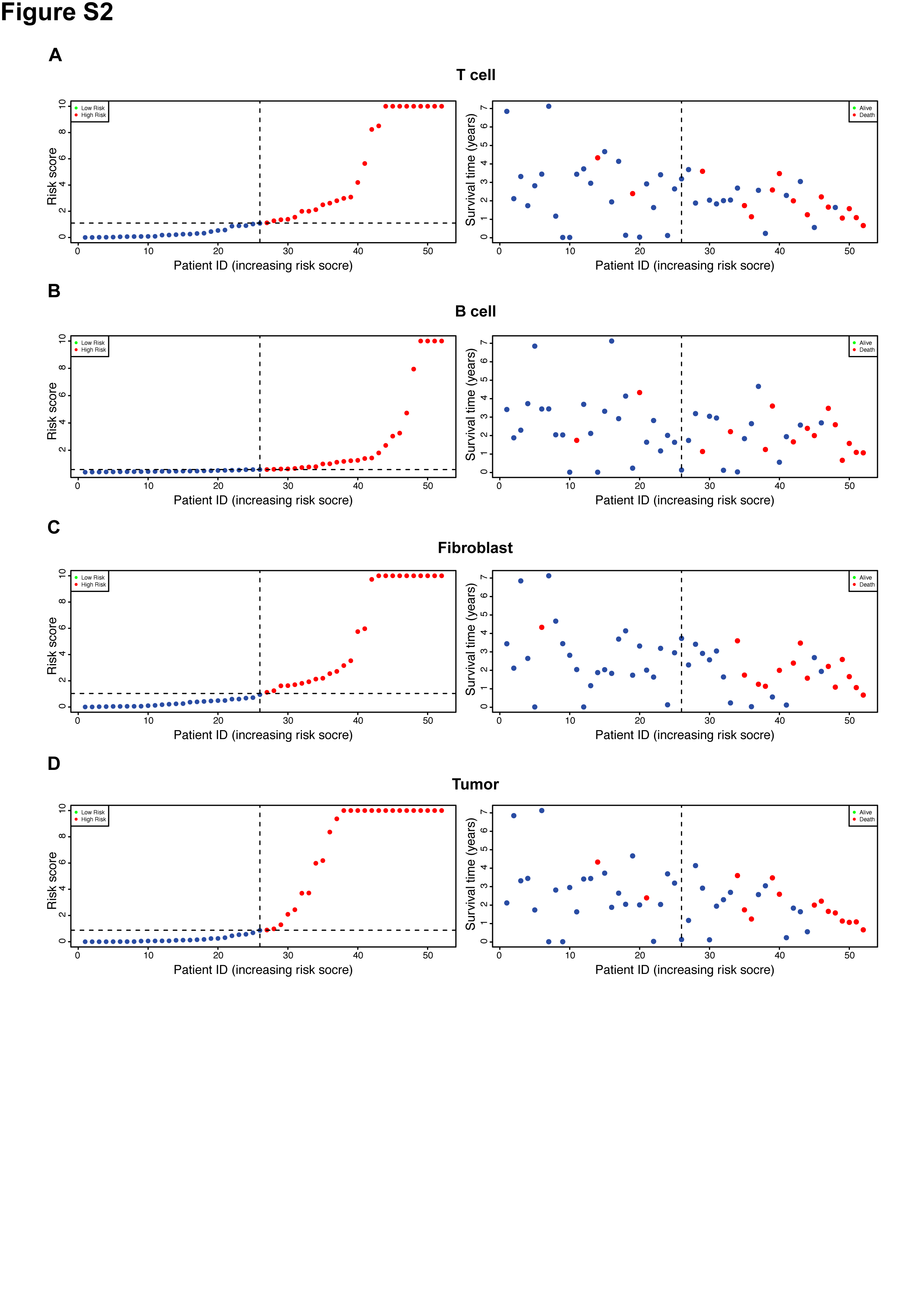

Supplement: Supplementary Figure 2 — Survival Analysis for Evaluating the Accuracy of the Gene Signature. (A) T cells: Distribution of risk scores between the CSIRG-high-risk and CSIRG-low-risk groups (left) and survival status of UVM patients in the CSIRG-high-risk and CSIRG-low-risk groups (right). Red dots represent CSIRG-high-risk patients who died, whereas green dots represent CSIRG-low-risk patients who survived. The survival distribution plot indicates that higher risk scores are associated with shorter overall survival. (B) B cells: Distribution of risk scores between the CSIRG-high-risk and CSIRG-low-risk groups (left) and survival status of UVM patients in the CSIRG-high-risk and CSIRG-low-risk groups (right). (C) Fibroblasts: Distribution of risk scores between the CSIRG-high-risk and CSIRG-low-risk groups (left) and survival status of UVM patients in the CSIRG-high-risk and CSIRG-low-risk groups (right). (D) Tumor cells: Distribution of risk scores between the CSIRG-high-risk and CSIRG-low-risk groups (left) and survival status of UVM patients in the CSIRG-high-risk and CSIRG-low-risk groups (right). The survival distribution plot indicates that higher risk scores are associated with shorter overall survival. [file Image2.tif]

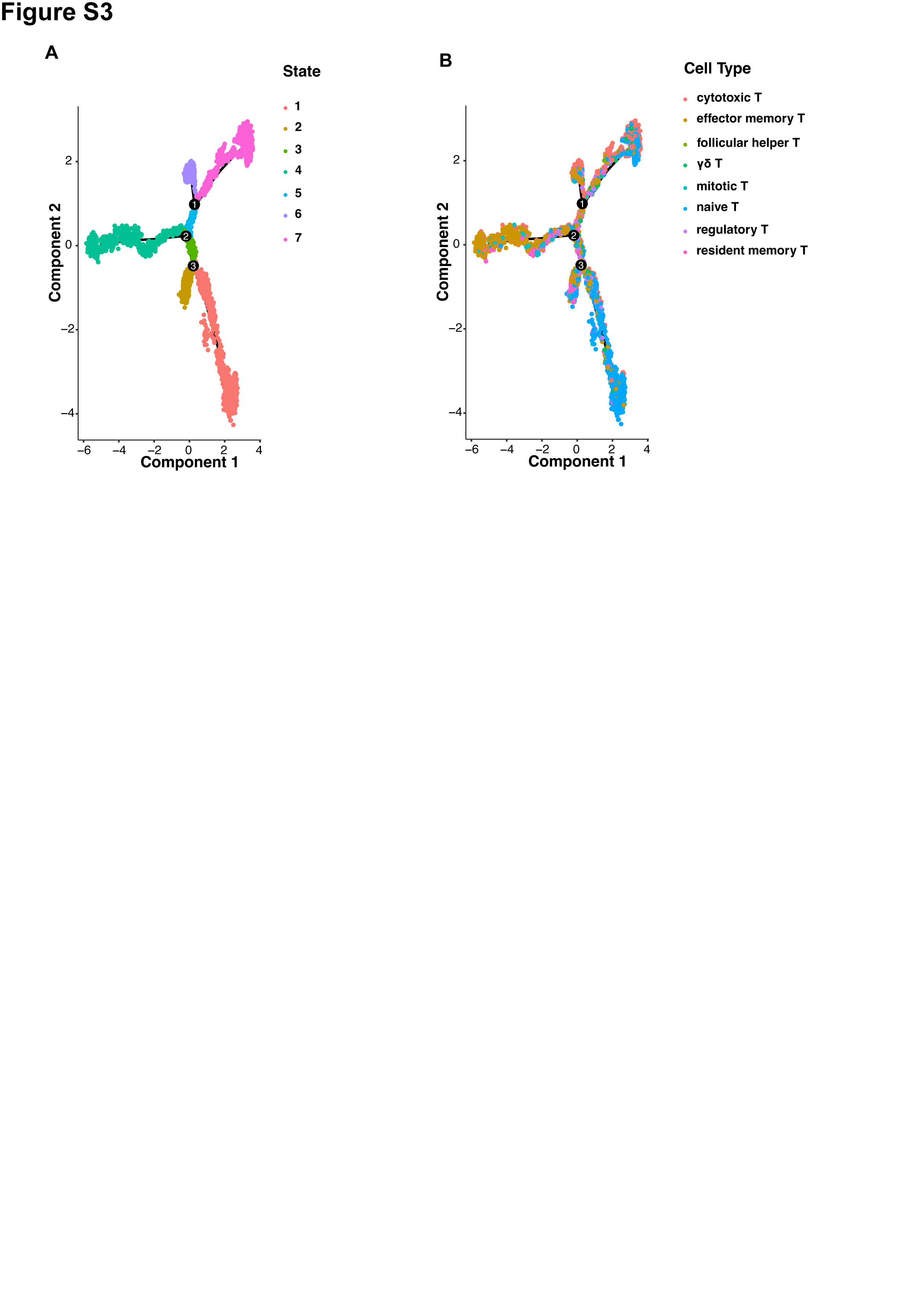

Supplement: Supplementary Figure 3 — (A) Pseudotime trajectory analysis of T cell subtypes utilizing the Monocle package, illustrating the ordering of T cells along a primary developmental trajectory with three distinct bifurcations. (B) The analysis revealed that the developmental hierarchy of T cells, commencing with naive T cells and advancing toward effector memory T cells and cytotoxic T cells, as determined by the pseudotime trajectory analysis. [file Image3.tif]

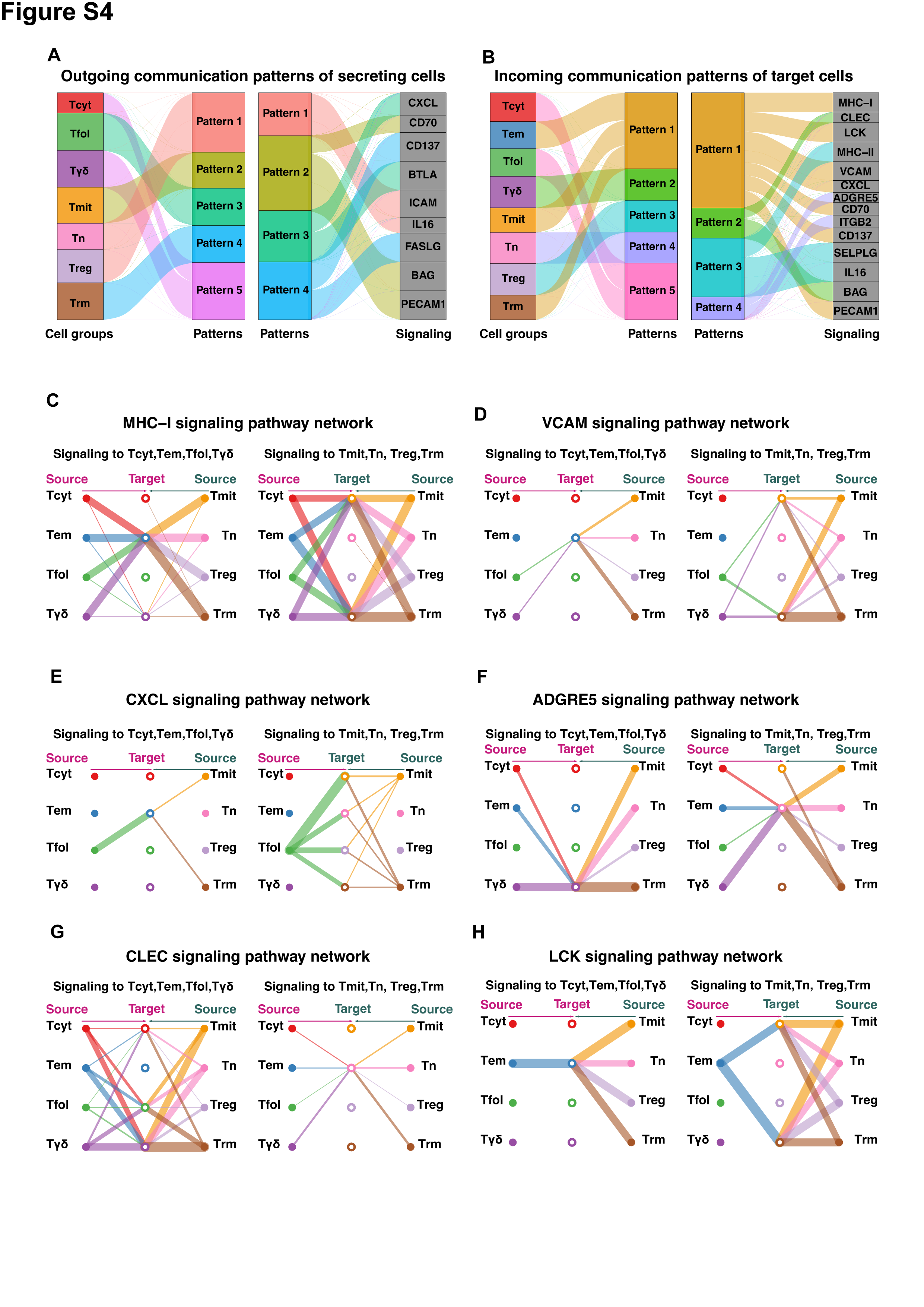

Supplement: Supplementary Figure 4 — Cell Communication Patterns and Signaling Pathways. (A) The communication patterns of outgoing cells in UVM, with flow widths indicating the contribution of each element to the pattern. Patterns 1-5 are represented, with specific pathways associated with naive T, regulatory T, mitotic T, follicular helper T, resident memory T, and γδ T cells. (B) The communication patterns of incoming cells in UVM, highlighting the pathways through which effector memory T, mitotic T, and resident memory T cells receive ligand stimulation, as well as γδ T and Treg cells. (C-H) the autocrine and paracrine signaling of key pathways (MHC-I, VCAM, CXCL, ADGRE5, CLEC, LCK) in T cells, with left panels showing the impact on various T-cell subtypes and right panels depicting signaling modulation among mitotic T, naive T, Treg, and resident memory T cells. Circle sizes and line widths correspond to cell numbers and communication strength, respectively. Abbreviations: Tcyt, Cytotoxic T; Tfol, Follicular helper T; Tn, Naive T; Treg, Regulatory T; Tem, Effector memory T; γδ T, Tγδ; Tmit, Mitotic T; Trm, Resident memory T. [file Image4.tif]
